# Inference of Gene Flow between Species from Genomic Data When the Mode, Direction, and Lineages are Misspecified

**DOI:** 10.1093/molbev/msaf121

**Published:** 2025-06-27

**Authors:** Yuttapong Thawornwattana, Tomáš Flouri, James Mallet, Ziheng Yang

**Affiliations:** Department of Organismic and Evolutionary Biology, Harvard University, Cambridge, MA 02138, USA; Department of Genetics, Evolution, and Environment, University College London, Gower Street, London WC1E 6BT, UK; Department of Genetics, Evolution, and Environment, University College London, Gower Street, London WC1E 6BT, UK; Department of Organismic and Evolutionary Biology, Harvard University, Cambridge, MA 02138, USA; Department of Genetics, Evolution, and Environment, University College London, Gower Street, London WC1E 6BT, UK

**Keywords:** gene flow, introgression, migration, multispecies coalescent, model misspecification, Bpp

## Abstract

Thanks to genomic data, interspecific gene flow is increasingly recognized as a major evolutionary force that shapes biodiversity. Two models have been developed in the multispecies coalescent (MSC) framework to infer gene flow from genomic data, assuming either constant-rate continuous migration (MSC-M) or discrete introgression/hybridization (MSC-I). The extreme simplicity of these models raises concerns about their usefulness as they represent misspecified models when applied to real data. Here, we study inference of gene flow under the MSC-M model, considering mis-assignment of gene flow onto incorrect parental or daughter lineages, misspecification of the direction of gene flow, and misspecification of the mode of gene flow. Mis-assignment of gene flow to an incorrect lineage causes large biases in the estimated rates. The Bayesian test has high power for inferring both recent and ancient gene flow, between either sister lineages or nonsister lineages, although misspecification of the direction of gene flow may make it hard to distinguish early divergence with gene flow from recent complete isolation. Misspecification of the mode of gene flow (MSC-I versus MSC-M) has small local effects, and gene flow is detected with high power despite the misspecification. We analyze a genomic dataset from the purple cone spruce (*Picea* spp., Pinaceae), which putatively arose through homoploid hybrid speciation, to demonstrate practical implications of our theoretical analyses. Overall, we find that the extremely idealized models of gene flow (in particular the discrete MSC-I model) are very effective for extracting information about species divergence and gene flow from genomic data.

## Introduction

Gene flow between species is an important process that shapes biodiversity we observe today. In the past two decades, genomic data have been widely used to detect gene flow and have considerably enriched our understanding of the role of gene flow in speciation and adaptation (Mallet et al. 2016). Commonly used methods for detecting gene flow and estimating its rate from genomic data are approximate, based on species triplets (or quartets if an outgroup is used) and/or rely on summaries of sequence data, such as genome-wide site pattern counts (e.g. the *D*-statistic and Hyde, Green et al. 2010; Kubatko and Chifman 2019), estimated gene tree topologies (e.g. Snaq, Solis-Lemus and Ane 2016; Jackson et al. 2017), or joint site frequency spectra (e.g. *δ*a*δ*i and Fastsimcoal2, Gutenkunst et al. 2009; Excoffier et al. 2021). Those methods do not make a full use of information in the data and often have reduced power to detect gene flow (Flouri et al. 2020; Ji et al. 2023; Pang and Zhang 2024; Ji et al. 2025). For example, most triplet summary methods cannot infer gene flow between sister lineages or identify the direction of gene flow (Jiao et al. 2021; Huang et al. 2022; Ji et al. 2023).

In this work, we focus on full-likelihood methods of inference under the multispecies coalescent (MSC) model, extended to account for gene flow (Rannala and Yang 2003; Jiao et al. 2021), applied to sequence data. Two approaches are commonly used for generating multilocus data, (i) sampling of short genomic fragments from sequenced genomes (e.g. Finger et al. 2022; Thawornwattana et al. 2022) and (ii) targeted sequence capture generating the so-called reduced representation data, including RADseqs (Andrews et al. 2016; Leaché and Oaks 2017), exome or transcriptome sequencing, ultraconserved elements (UCEs, Faircloth et al. 2012), anchored hybrid enrichment (AHE, Lemmon et al. 2012), conserved nonexonic elements (CNEEs, Edwards et al. 2017), and rapidly evolving long exon capture (RELEC, Karin et al. 2020). We refer to genomic fragments generated using either strategy as loci (irrespective of whether they are protein-coding). The MSC model assumes no recombination within each locus and free recombination between loci (see Zhu et al. 2022; Yan et al. 2023 for simulations that examine the impact of recombination on inference under the MSC models).

**Fig. 1.**
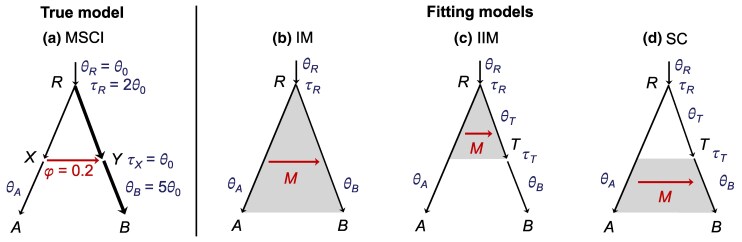
a) An MSC-I model for two species (A,B), with introgression from *A* to *B* at time $\tau_{X}$ with introgression probability φ, used to generate data. We assume that population sizes of *A* and *B* do not change at the time of introgression (i.e. $\theta_{A}=\theta_{X},\theta_{B}=\theta_{Y}$). Thus, the parameter vector is $\theta_{i}=\{\varphi ,\tau_{X},\tau_{R},\theta_{A},\theta_{B},\theta_{R}\}$. Each branch has an associated population size parameter θ=4Nμ, where *N* is the effective population size and *μ* is the mutation rate per site per generation. Time is measured as the expected number of mutations per site, with τ=Tμ, where *T* is the divergence time in generations. Parameter values used in simulation are as shown: $\theta_{A}=\theta_{X}=\theta_{R}=\theta_{0}$ (thin branches), $\theta_{B}=\theta_{Y}=5\theta_{0}$ (thick branches), $\tau_{X}=\theta_{0}$, $\tau_{R}=2\theta_{0}$ and φ=0.2, with $\theta_{0}=0.002$. b–d) Three MSC-M models used to analyze the data: IM (isolation with migration), IIM (isolation with initial migration), and SC (secondary contact). Gray shading indicates a period of continuous gene flow from *A* to *B* at rate $M_{AB}=N_{B}m_{AB}\equiv M$ migrants per generation, where $m_{AB}$ is the proportion of migrants in *B* from *A* per generation. The parameter vector of the IM model is $\theta_{\mathrm{IM}}=\{M,\tau_{R},\theta_{A},\theta_{B},\theta_{R}\}$, while those for IIM and SC are $\theta_{\mathrm{IIM}}=\theta_{\mathrm{SC}}=\{M,\tau_{R},\tau_{T},\theta_{A},\theta_{B},\theta_{T},\theta_{R}\}$. The IIM and SC models are implemented in Bpp as instances of the MSC-M model by including an unsampled ghost species that is sister to *B* and diverged with *B* at time $\tau_{T}$. This creates two *θ* parameters for branches *RT* and *TB* as the current version of Bpp does not implement the constraint $\theta_{T}=\theta_{B}$.

Two idealized modes of gene flow have been modeled in the MSC framework (Jiao et al. 2021). First, in the MSC-with-introgression (MSC-I) model (Flouri et al. 2020), also known as the multispecies network coalescent (MSNC; Yu et al. 2012; Wen et al. 2016; Wen and Nakhleh 2018; Zhang et al. 2018) or network multispecies coalescent (NMSC) model (Ané et al. 2024), gene flow is a discrete event and occurs in a pulse at a specific time point. The amount of gene flow is measured by the introgression probability, $\varphi_{A\to B}$, which represents the proportion of migrants in *B* from *A* at the time of introgression (Fig. 1a).

Second, the MSC-with-migration (MSC-M) model (Flouri et al. 2023) assumes that gene flow is continuous and occurs at a constant rate per generation over an extended time period. Gene flow from populations *A* to *B* is measured by the population migration rate $M_{A\to B}=N_{B}m_{A\to B}$, which is the expected number of individuals in *B* that are migrants from *A* per generation, with $N_{B}$ to be the effective population size of *B*, and $m_{A\to B}$ the proportion of individuals in *B* that have migrated from *A*. Note that we use the real-world view with time running forward when defining gene-flow parameters. The MSC-M model includes the isolation-with-migration (IM) model (Nielsen and Wakeley 2001; Hey and Nielsen 2004; Hey 2010; Zhu and Yang 2012; Dalquen et al. 2017; Hey et al. 2018; Jones 2019) (Fig. 1b). Variants of MSC-M models include the isolation-with-initial-migration (IIM) model (Fig. 1c; Costa and Wilkinson-Herbots 2017) and the secondary contact (SC) model (Fig. 1d; Costa and Wilkinson-Herbots 2021). In this paper we use the terms “introgression” to refer to pulse gene flow in the MSC-I model and “migration” for continuous gene flow in the MSC-M model.

The MSC-I and MSC-M models may be viewed as extreme special cases of a general model with variable rates of gene flow over time. In the real world, the rate of gene flow may be expected to vary over time as the geographical distribution of species expand or shrink, impacting their opportunities to meet and hybridize, and as the intensity of natural selection purging introgressed alleles fluctuates over time, influenced by multiple factors including recombination and genetic drift (Martin and Jiggins 2017; Moran et al. 2021). While both the MSC-I and MSC-M models are wrong when applied to genomic data, it is interesting to know whether they produce similar inferences of gene flow (e.g. the lineages and direction of gene flow) or similar estimates of key population parameters such as species divergence times and rates of gene flow. How robust are parameter estimates to the assumed mode of gene flow? Similarly will we infer gene flow if the introgression events are incorrectly assigned to parental or daughter lineages to lineages genuinely involved in gene flow? Will we detect gene flow if the direction of gene flow is misspecified?

Here we address those questions. We investigate the impact of three kinds of model specifications on Bayesian inference of gene flow using genomic data:

1. The mode of gene flow might be incorrectly assumed. For instance, gene flow might have occurred as a single pulse of introgression or hybridization (MSC-I) but continuous gene flow (MSC-M) is assumed in data analysis.
2. Lineages involved in gene flow may be incorrectly specified. Currently, it is very challenging to assign gene-flow events to branches in a species phylogeny (Pang and Zhang 2024). For example, when gene flow is detected in many species triplets and is assigned to ancestral branches using criteria such as *f*-branch (Malinsky et al. 2018), it may be assigned incorrectly to parental or daughter branches (Suvorov et al. 2022; Ji et al. 2023; Thawornwattana et al. 2023b).
3. The direction of gene flow may be misspecified. Indeed, most summary methods that rely on gene tree topologies or site-pattern counts cannot identify the direction of gene flow (Hibbins and Hahn 2022).

In this paper, we do not consider the scenario of ghost introgression involving a source population that has gone extinct or is not sampled in the data (Huang et al. 2022; Tricou et al. 2022; Pang and Zhang 2023, 2024). Furthermore, we do not consider the search in the space of all possible models of gene flow given a set of species, either with or without the species tree fixed. Currently, inference of gene-flow models is a challenging task for both summary and full likelihood methods. While MCMC algorithms are implemented to update the MSC-I model in the programs Phylone/mcmc-seq (Wen and Nakhleh 2018) and *beast (Zhang et al. 2018), the implementations are computationally unfeasible except for very small datasets with <100 loci. The programs *δ*a*δ*i (Gutenkunst et al. 2009) and Fastsimcoal2 (Excoffier et al. 2021) can be used to compare candidate gene-flow models. These methods use data of joint site frequency spectra at SNP sites, which are genome-wide averages, and ignore information in the variation in genealogical relationships across the genome. Such data have fundamental limits in information content when used to infer the demographic history of one species (Terhorst and Song 2015; Baharian and Gravel 2018). A recent paper demonstrated that surprisingly commonly used summary methods such as the *D*-statistic (Green et al. 2010), Hyde (Kubatko and Chifman 2019), Snaq (Solis-Lemus and Ane 2016; Jackson et al. 2017), and Phylonet/mpl (Yu et al. 2012; Yu and Nakhleh 2015) do not have the capability to distinguish among different models (such as inflow, outflow, and ghost introgression); in other words, the different gene-flow models are unidentifiable by these methods (Pang and Zhang 2024). Currently, choice of gene-flow models is an area of active research.

Thus, we limit the scope of our study to the three kinds of model specifications identified above. We characterize the power and false positives of Bayesian tests of gene flow, and bias in estimation of the rate of gene flow under model misspecification. We use asymptotic analysis to deal with infinite data under simple scenarios and computer simulation to consider finite datasets under more complex scenarios. We corroborate our theoretical analyses by analyzing a genomic dataset from the purple cone spruce under the MSC-I and MSC-M models. We use the Bayesian program Bpp because of its computational efficiency (Flouri et al. 2018, 2023), but the results should apply to other full-likelihood methods as well (e.g. Ima3, Hey et al. 2018). Because full-likelihood methods utilize all information in the data concerning the model and parameters, whereas summary methods make use of only a portion of that information, our results should also shed light on the behaviors and limitations of summary methods under similar situations. This work complements our previous studies on model misspecification where the MSC-I model was assumed in analysis of data generated under the MSC-M model (Jiao et al. 2020; Huang et al. 2022) or when the direction of introgression was misspecified under the MSC-I model (Thawornwattana et al. 2023a). We summarize results from both this study and the previous studies in the Conclusions section.

## Results and Discussion

### The Case of Two Species

Suppose that gene flow occurs from *A* to *B* at time $\tau_{X}$ with introgression probability φ (Fig. 1a) but we analyze the data under MSC-M models assuming continuous migration over an extended time period (Fig. 1b–d). What will the estimate of the migration rate (*M*) be like? Will we detect gene flow despite misspecification of the mode of gene flow? Similarly, what are the effects of misspecified direction of gene flow? We approach these questions using a combination of asymptotic analysis (as the number of loci approaches infinity) and computer simulation, following Jiao et al. (2020) and Huang et al. (2022). The asymptotic analysis is tractable in the simple case of two species with one sequence per species per locus, while simulations can be performed for any number of species, any number of sequences per species, and any finite number of loci.

#### Asymptotic Theory in the Two-species Case

We develop an asymptotic theory of maximum-likelihood (ML) estimation for the simple model of gene flow for two species, with introgression from *A* and *B* (Fig. 1a), and with data of two sequences (one from each species) per locus. The true model is MSC-I, with parameter vector $\theta_{i}$ (Fig. 1a), and the data are analyzed to estimate the parameter vector $\theta_{m}$ under three variants of the MSC-M model: isolation with migration (IM), isolation with initial migration (IIM), and SC (Fig. 1b–d). Note that when we define parameters of introgression probability or migration rate time runs forward. As there is only one sequence per species per locus, $\theta_{B}$ is not used in MSC-I, and $\theta_{B}$ and $\theta_{T}$ are unidentifiable under any of the MSC-M models. Thus, the parameter vector for the true MSC-I model is $\theta_{i}=\{\varphi ,\tau_{X},\tau_{R},\theta_{A},\theta_{R}\}$ (Fig. 1a), while that for the fitting MSC-M model is $\theta_{\mathrm{IM}}=\{M,\tau_{R},\theta_{A},\theta_{R}\}$ or $\theta_{\mathrm{IIM}}=\theta_{\mathrm{SC}}=\{M,\tau_{T},\tau_{R},\theta_{A},\theta_{R}\}$ (Fig. 1b–d). Later, we analyze simulated data with multiple sequences sampled per species per locus, which allow estimation of the full parameter set (Fig. 1).

Consider an infinite number of loci, each with two sequences (*a* and *b*) of length *n*. We assume the infinite-sites mutation model, so that the sequence data at each locus is summarized as *x* differences out of *n* sites. The probability for *x* is given by averaging over the unobserved coalescent time *t* between the two sequences,


(1) $$f(x;\theta )=\int_{0}^{\infty }f(x\;|\;t)f(t;\theta )\;\;dt.$$

The density of coalescent time, f(t;θ), depends on the model of gene flow, and is given in SI text for the MSC-I model of Fig. 1a and the MSC-M models of Fig. 1b–d. Given the coalescent time *t*, the expected number of mutations at the locus is n×2t, so that f(x|t) is given by the Poisson probability


(2) $$f(x\;|\;t)=\frac{1}{x!}(2nt{)}^{x}{\text{e}}^{-2nt}.$$

This leads to a closed-form expression of f(x;θ), as in Huang et al. (2022).

Under the MSC models, data at different loci are independently and identically distributed (i.i.d.). When the number of loci L→∞, the MLE ${\hat{\theta }}_{m}$ under the wrong MSC-M model converges to $\theta_{m}^{*}$, which minimizes the Kullback–Leibler (KL) divergence


(3) $$D(\theta_{i}∥\theta_{m})=\sum_{x=0}^{n}f_{i}(x;\theta_{i})\log\;\frac{\;f_{i}(x;\theta_{i})}{f_{m}(x;\theta_{m})}.$$

Here the subscripts “i” and “m” specify the model under which the parameters and probabilities are defined. In effect $\theta_{i}$ in MSC-I is fixed and $f_{i}(x;\theta_{i})$ represents the data while $f_{m}(x;\theta_{m})$ represents the fitting model, and $\theta_{m}$ is estimated by minimizing *D*. The estimate $\theta_{m}^{*}$ is known as the *pseudo-true parameter value* or the *best-fitting parameter value* under the fitting MSC-M model. Note that when L→∞, the Bayesian estimate (the posterior mean) approaches the same limit ($\theta_{m}^{*}$) as the ML estimate (MLE). Because of model mismatch, a perfect fit is impossible, so that D>0.

Of particular interest is the correspondence between the introgression probability φ in MSC-I and the migration rate *M* in MSC-M. Under MSC-M, the probability that a lineage from species *B* traces back to *A* (irrespective of the migration time), when one traces the genealogical history of the sampled sequences backwards in time, is


(4) $$\varphi_{0}=1-e^{-\frac{4M}{\theta_{B}}\Delta \tau },$$

where Δτ is the time period of migration (Huang et al. 2022). At the mutational time scale used here, migration occurs at the Poisson rate of $4M/\theta_{B}$, and equation (4) is the cumulative distribution function of the exponential waiting time until migration. Note that the introgression probability φ in MSC-I is also the probability that a lineage from species *B* traces back to *A* (at time $\tau_{X}$). Thus both φ in MSC-I and $\varphi_{0}$ in MSC-M measure the expected total amount of gene flow.

Inverting equation (4) gives


(5) $$M_{0}=\frac{\theta_{B}}{4\Delta \tau }\log\;\left(\frac{1}{1-\varphi }\right).$$

#### The Limiting Values of the MLEs in the Two-species Case

We use the above theory to study the asymptotic behavior of parameter estimation under the MSC-M models of Fig. 1b–d when the data are generated under the MSC-I model (Fig. 1a). We used two population sizes on the species tree, $\theta_{0}=0.002$ for the thin branches and $\theta_{1}=0.01$ for the thick branches (Fig. 1a). The species divergence time is $\tau_{R}=2\theta_{0}$ while introgression occurs at time $\tau_{X}=\theta_{0}$. In our simulation, the divergence times (*τ*s) and population-size parameters (*θ*s) are proportional. This mimics the use of different types of genomic data with different neutral mutation rates (e.g. exons versus noncoding DNA). We varied the sequence length (*n*) and the introgression probability (φ).

**Fig. 2.**
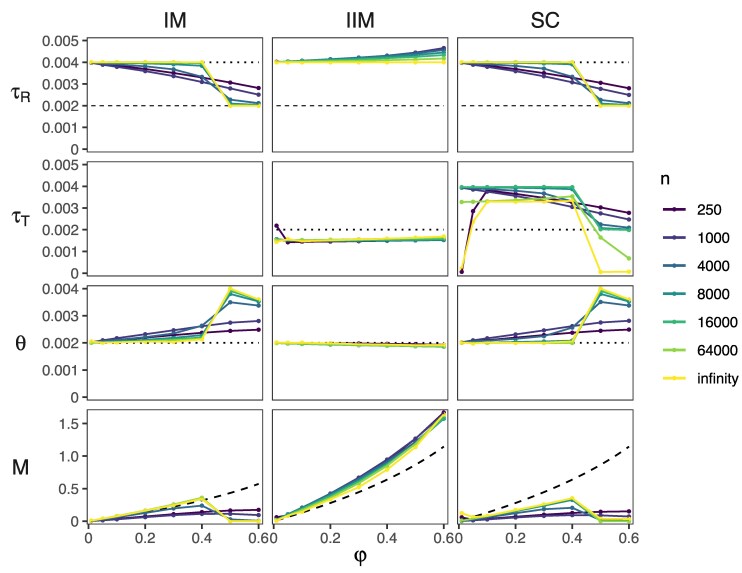
Best-fitting parameter values from the asymptotic analysis under the IM, IIM and SC models (Fig. 1b–d) of data generated under the MSC-I model (Fig. 1a). In effect, the dataset consists of infinitely many loci, each with two sequences (one from each species) of *n* sites. The two population sizes, $\theta_{A}$ and $\theta_{R}$, were constrained to be equal, denoted by *θ*. Horizontal dotted lines indicate true values. For $\tau_{R}$, the dashed line indicates the introgression time $\tau_{X}$ in the MSC-I model. For *M*, dashed curves indicate the expected value $M_{0}$ based on equation (5), calculated using the true $\theta_{B}$ and the expected time duration of migration (Δτ), which is $\tau_{R}$ for IM, $\tau_{R}-\tau_{X}$ for IIM, and $\tau_{X}$ for SC. The true and best-fitting distributions of the coalescent time (*t*) are in supplementary fig. S1, Supplementary Material online.

The limits of the MLE ($\theta_{m}^{*}$, equation (3)) are shown in Fig. 2. The true and best-fitting distributions of the coalescent time $t_{ab}=t$ are in supplementary fig. S1, Supplementary Material online, with the achieved KL values in supplementary fig. S2, Supplementary Material online.

Among the three MSC-M models of Fig. 1b–d, the IIM model provide the most sensible parameter estimates, with $M^{*}$ tracking the introgression probability (φ) in the MSC-I model (Fig. 2) and with the lowest KL divergence (supplementary fig. S2, Supplementary Material online). The estimates $\tau_{R}^{*}$ and $\theta_{R}^{*}=\theta_{A}^{*}$ also match the true values. This means coalescence in the ancestral population (*R*) is correctly accounted for. The time at which migration stops ($\tau_{T}^{*}$) is slightly younger than the actual introgression time ($\tau_{T}^{*}=0.0015$–0.0016 $<\tau_{X}=0.002$). Under the MSC-I model, gene flow results in a peak in the density of the coalescent time $t_{ab}$ at $\tau_{X}$ (supplementary fig. S1, Supplementary Material online, black curve). By contrast, coalescence due to migration under the IIM model peaks in the middle of the migration period (supplementary fig. S1, Supplementary Material online, dark blue curve, middle column). Thus having $\tau_{T}^{*}<\tau_{X}$ gives a better fit.

The IM and SC models produce similar best-fitting parameter values, different from IIM (Fig. 2). Under the SC model, the time at which migration starts ($\tau_{T}^{*}$) is often close to the divergence time ($\tau_{R}^{*}$), making it similar to the IM model. At small values of φ, the estimated migration rate $M^{*}$ under IM and SC matches closely the expected value $M_{0}$ (calculated using equation (5) using the migration period 0–$\tau_{R}$ and the true population size $\theta_{B}$; Fig. 2, first column) and the species divergence time $\tau_{R}^{*}$ matches the true value as the number of sites approaches infinity. At large φ (say φ>0.5), *M* is seriously underestimated, accompanied by an underestimation of $\tau_{R}$ (with $\tau_{R}^{*}\approx \tau_{X}$) and an overestimation of *θ* (Fig. 2 and supplementary fig. S1, Supplementary Material online).

Both IM and SC assume continuous and ongoing migration and, at a high *M*, predict presence of recent coalescence with $t_{ab}\approx 0$ or nearly identical sequences from the two species. In the data (generated under the MSC-I model with all migration occurring at time $\tau_{X}>0$), such recent coalescence with $t_{ab}\approx 0$ is absent and nearly identical sequences between species are uncommon. The rarity of nearly identical sequences between species in the data is then hard to reconcile with the IM and SC models with a high migration rate, especially when the sequence is long (large *n*) (Fig. 2 and supplementary fig. S1, Supplementary Material online). As a result, the models underestimate both *M* and $\tau_{R}$, and in effect explain recent coalescence (small $t_{ab}$ due to introgression at time $\tau_{X}$) as coalescence in the common ancestor *R* (with $\tau_{R}^{*}\approx \tau_{X}$).

#### Simulation Results in the Two-species Case

**Fig. 3.**
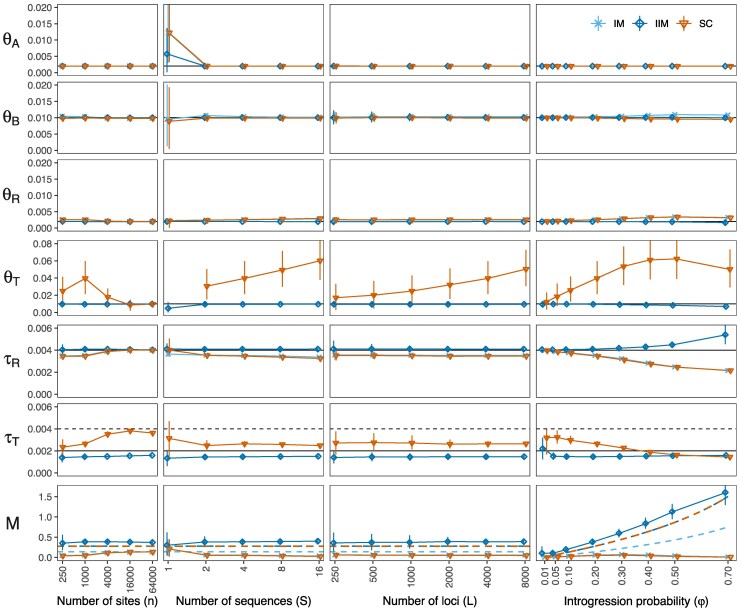
Parameter estimates under the three MSC-M models (IM, IIM, and SC; Fig. 1b–d) obtained from Bpp analysis of data generated under the MSC-I model (Fig. 1a), summarized as posterior means and 95% HPD CIs averaged over 30 replicate datasets. In the base case, each dataset consists of L=4,000 loci, with S=4 sequences per species at each locus and n=1,000 sites per sequence. Parameters in the MSC-I model are given in the legend to Fig. 1. We varied four factors one at a time, keeping other factors fixed at the base case: the number of sites per sequence (*n*), the number of sequences per species (*S*), the number of loci (*L*), and the introgression probability (φ). The parameters $\theta_{T}$ and $\tau_{T}$ are specific to the IIM and SC models. When S=1, $\theta_{B}$ is unidentifiable in the IIM model (Fig. 1c), and $\theta_{T}$ is unidentifiable in the SC model (Fig. 1d). Horizontal solid lines indicate the true values in the MSC-I model used to generate the data. For $\tau_{T}$, the horizontal dotted line indicates $\tau_{R}$, the upper limit of $\tau_{T}$. For *M*, a dashed curve indicates the expected value $M_{0}$ based on equation (5), assuming the true $\theta_{B}$ and the expected duration of migration Δτ; see legend to Fig. 2. The *x*-axes for *n*, *S*, and *L* are on a logarithmic scale.

The asymptotic analysis assumes one sequence per species per locus. To accommodate multiple sequences per species, we used simulation. Data are simulated under the MSC-I model (Fig. 1a) and analyzed under the MSC-M models (IM, IIM, and SC; Fig. 1b–d), using Bpp. The Markov chain Monte Carlo (MCMC) algorithm in Bpp averages over the gene genealogy underlying the sequence alignment at each locus, similar to the integration in equation (1) averaging over the coalescent time *t* in the case of two sequences. We assume the JC mutation model (Jukes and Cantor 1969). In the base case, each dataset consisted of L=4,000 loci, with S=4 sequences per species per locus, and n=1,000 sites per sequence, and the introgression probability is set at φ=0.2. Then we varied the number of sites per sequence (*n*), the number of sequences per species (*S*), the number of loci per species (*L*), and the introgression probability (φ). The first three factors are related to data size while the last is a parameter that measures the amount of gene flow. We are interested in how these factors influence posterior estimates of parameters, in particular the migration rate *M*. The posterior means and 95% HPD CIs for parameters in the MSC-M models are summarized in Fig. 3, while the true and best-fitting distributions of the coalescent times ($t_{ab},t_{aa},t_{bb}$) are in supplementary figs. S3–S6, Supplementary Material online.

First, we consider the sequence length (*n*; Fig. 3, first column). This has little impact on the posterior means and highest-probability-density (HPD) credibility intervals (CIs) for parameters $\theta_{A}$, $\theta_{B}$, $\theta_{R}$, and $\tau_{R}$ under all three models, or on $\theta_{T}$ and $\tau_{T}$ under the IIM model. Even with short sequences (n=250), those parameters are precisely estimated. However, use of longer sequences improves the precision in the estimated migration rate *M* under the IIM model. At n=64,000 sites, the estimate is $\hat{M}=0.37$, close to the limiting value $M^{*}=0.33$ from our asymptotic analysis for data of infinitely many loci of two sequences under the assumption of equal population sizes (see above). Under the IM or SC models, we obtained much smaller estimates, with $\hat{M}$ increasing from 0.05 at n=250 to 0.13 at n=64,000, accompanied by an increase in ${\hat{\tau }}_{T}$ in the SC model, which converges to $\tau_{R}$ at large values of *n* (Fig. 3; also see supplementary fig. S3, Supplementary Material online). Thus, the SC model converges to the IM model when n→∞. Those results agree with our asymptotic results (Fig. 2). The increased rate ($\hat{M}$) and duration (${\hat{\tau }}_{T}$) of gene flow in the SC model with the increase of *n* may be explained by the increasingly stronger evidence of gene flow in longer sequences. The large $\hat{M}$ also improves ${\hat{\theta }}_{T}$ because migration helps explain large variation in the recipient population *B* caused by introgression from *A* in the true MSC-I model (see the improved fit to the coalescent time $t_{bb}$ at large *n* in supplementary fig. S3, Supplementary Material online).

Under the IIM model, the time when migration ends ($\tau_{T}$) is estimated to be about 0.0015, as predicted by our asymptotic analysis of both finite and infinitely long sequences (Fig. 2), while the true introgression time $\tau_{X}$ is 0.002. One might expect ${\hat{\tau }}_{T}$ (the time at which the migration period ends) to converge to $\tau_{X}$ since this is the smallest time at which sequences from *A* and *B* can coalesce (i.e. the smallest $t_{ab}$). We obtain ${\hat{\tau }}_{T}<\tau_{X}$. This may be partly attributable to the difference in how the MSC-I and the IM models account for the reduced $t_{ab}$ due to gene flow. The probability density of $t_{ab}$ peaks at the introgression time $\tau_{X}$ in the MSC-I model while it peaks in the middle of the migration period ($\tau_{T},\tau_{R}$) in the IIM model (supplementary fig. S1, Supplementary Material online). Thus having a migration period that ends after $\tau_{X}$ better accommodates $t_{ab}$ in the data. In summary, the IIM model is able to detect more gene flow and provides more precise and accurate estimates of population sizes and divergence times even with short sequences (n=250) while the IM and SC models require at least 4,000 sites per locus to be able to detect substantial amounts of gene flow (Fig. 3 for *M* against *n*).

Second, we consider the effects of the number of sequences per species (*S*; Fig. 3, second column). Overall, estimation under all three MSC-M models benefited considerably from including multiple samples per species (with S>1). With only one sequence per species (S=1), no coalescent events can occur in *B* and *T*, so that $\theta_{B}$ in IIM and $\theta_{T}$ in SC are unidentifiable, and other parameters such as $\theta_{A},\theta_{T},\tau_{T},M$ have large uncertainties as well, although $\tau_{R}$ and $\theta_{R}$ are well estimated. When S>1, all parameters in all three MSC-M models are identifiable, and furthermore, even those parameters that are identifiable at S=1 have much narrower CIs (except for $\theta_{T}$ in SC for the same reason as in the case of varying *n*). As before, the IIM model recovered more gene flow, with $\hat{M}=0.40$ (0.35, 0.45) at S=16, in comparison with $\hat{M}<0.1$ for IM and SC.

Third, the number of loci (*L*; Fig. 3, third column and supplementary fig. S5, Supplementary Material online) is the sample size in the model as data at different loci are i.i.d. Increasing *L* led to narrower CIs. In theory, the CI width should reduce by a half as *L* quadruples. This holds approximately for most parameters except for $\theta_{T}$ in the SC model, which is poorly estimated.

Lastly, we consider the impact of the amount of gene flow in the data (φ; Fig. 3, last column). For all analysis models, the extant ($\theta_{A},\theta_{B}$) and ancestral ($\theta_{R}$) population sizes are well-estimated, with the posterior mean close to the true value and with narrow CIs. Consistent with our asymptotic results, only the IIM model is able to estimate *M* that increases with φ. However, due to the model misspecification, as φ increases, the IIM model increasingly overestimate $\tau_{R}$ while ${\hat{\tau }}_{T}$ stays largely unchanged, resulting in an increasingly long period of migration (${\hat{\tau }}_{T},{\hat{\tau }}_{R}$). In effect deep coalescent events between sequences from *A* and *B* in *R* are being mis-interpreted as a result of migration after species divergence (supplementary fig. S6, Supplementary Material online). By contrast, the IM and SC models only detect small amounts of gene flow ($\hat{M}<0.1$) regardless of the true value of φ (Fig. 3). In the MSC-I model, larger values of φ lead to smaller $t_{ab}$, with a peak at the introgression time ($\tau_{X}$). The IM and SC models accommodate small $t_{ab}$ in the data as coalescence in the ancestral population, with ${\hat{\tau }}_{R}$ gradually decreasing from $\tau_{R}$ to $\tau_{X}$ as φ increases. This reduction in ${\hat{\tau }}_{R}$ is associated with an increase in ${\hat{\theta }}_{R}$. This pattern agrees with our asymptotic analysis (Fig. 2, n=1,000), which predicts that ${\hat{\tau }}_{R}\to \tau_{X}$ as n→∞ and L→∞.

Why do the IM and SC models detect much less gene flow than the IIM model across a wide range of values of φ? Those two models assume ongoing migration up to the present time and predict recent coalescent events between sequences from the two species (with $t_{ab}\approx 0$), but no such coalescence exists in the data or in the true MSC-I model. High migration rates (*M*) in the IM and SC models are thus incompatible with the data. Under the SC model, ${\hat{\theta }}_{T}$ is usually much larger than the true value and with a wide CI, and $\hat{M}$ is close to zero. This is because introgression from *A* into *B* in the data (generated under MSC-I) increases genetic variation in *B*. Here, with $\theta_{B}$ being well estimated and $\hat{M}$ being close to zero, having a large value of ${\hat{\theta }}_{T}$ helps explain genetic variation in *B*. This also explains why ${\hat{\theta }}_{T}$ peaks at φ=0.5, where genetic variation in *B* is the largest under the MSC-I model (Fig. 3).

In summary, the simulation results for the case of two species agree with our asymptotic analysis of data of an infinite number of loci with one sequence per species (Fig. 2; n=1,000). The IIM model provides the most sensible estimates and is able to recover approximately correct amounts of gene flow. However, $\tau_{R}$ is overestimated when φ is high. The IM and SC models are qualitatively similar: they can recover much less gene flow than the IIM model and require long sequences of at least n=4,000 sites per locus to detect a reasonable amount of gene flow. While increasing the data size in any way (n,S,L) helps with the information content, including multiple sequences per species (S>1) is particularly important.

*The Bayesian test of gene flow.* We also applied the Bayesian test of gene flow to analyze the simulated datasets of Fig. 3, with results shown in supplementary fig. S7, Supplementary Material online. The null hypothesis $H_{0}\;:\;M_{A\to B}=0$ and the alternative hypothesis $H_{1}\;:\;M_{A\to B}>0$ are compared using Bayes factors calculated using the Savage–Dickey density ratio (Ji et al. 2023). This formulation of the Bayes factor contrasts the prior and posterior probabilities that the migration rate is very low (M<ϵ=0.001) to assess the evidence in the data in support of gene flow. Overall, the test has high power, rejecting the null of no gene flow (with $B_{10}>100$) in almost all datasets when the number of sites (*n*), the number of sequences (*S*) and the number of loci (*L*) varied around the base case (supplementary fig. S7, Supplementary Material online). At very low introgression probability (φ=0.01, say), the test based on IM and SC has virtually no power while that based on IIM has full power. The situation is similar at very high introgression probabilities (φ=0.7, say). Note that the MSC-I model with φ=1 reduces to a model of complete isolation with no gene flow. Overall, the Bayesian test of gene flow, in particular the test based on the IIM model, has high power despite the misspecification of the mode of gene flow. Detecting gene flow through the test appeared to be a much easier task than estimating the amount of gene flow.

### The Case of Four Species

**Fig. 4.**
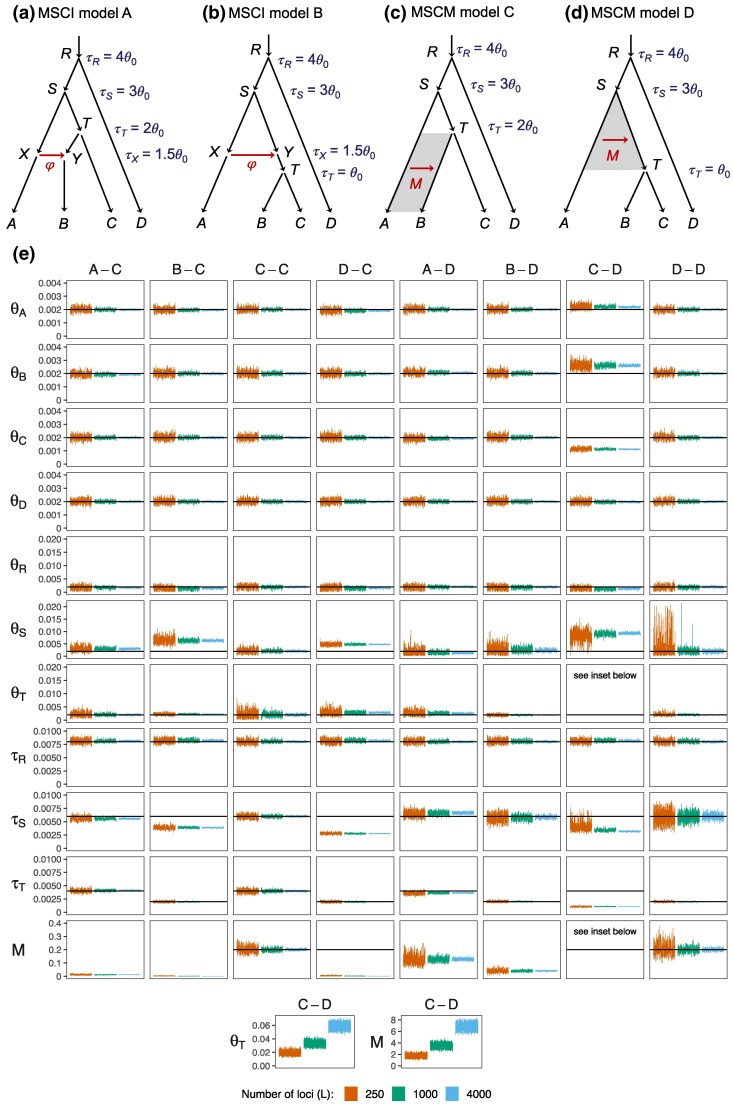
a–b) Two introgression (MSC-I) models and c–d) two migration (MSC-M) models used in simulation. All branches have population size $\theta_{0}=0.002$. In MSC-I model A, the species divergence and introgression times are $\tau_{R}=4\theta_{0}$, $\tau_{S}=3\theta_{0}$, $\tau_{T}=2\theta_{0}$, and $\tau_{X}=\tau_{Y}=1.5\theta_{0}$. In MSC-I model B, $\tau_{R}=4\theta_{0}$, $\tau_{S}=3\theta_{0}$, $\tau_{T}=\theta_{0}$, and $\tau_{X}=\tau_{Y}=1.5\theta_{0}$. Introgression probability is φ=0.2. In MSC-M model C, $\tau_{R}=4\theta_{0}$, $\tau_{S}=3\theta_{0}$, and $\tau_{T}=2\theta_{0}$, with migration occurring from species *A* to *B* during ($0,\tau_{T}$) at rate M=0.2 migrants per generation. In MSC-M model D, $\tau_{R}=4\theta_{0}$, $\tau_{S}=3\theta_{0}$, and $\tau_{T}=\theta_{0}$, with migration from *A* to *T* during ($\tau_{T},\tau_{S}$) at rate M=0.2. e) The 95% HPD CIs of parameters from 100 replicate datasets of L=250,1,000, and 4,000 loci. Column labels refer to the simulation model followed by the analysis model; e.g. “A-C” means the data were simulated under model A and analyzed under model C. Black solid lines indicate the true value. Estimates of $\theta_{T}$ and *M* for the C-D setting are shown separately at the bottom due to their extreme values.

We extend our simulation to more complex cases of four species with the phylogeny ([*A*, *B*, *C*], *D*), in which *D* is an outgroup, not involved in gene flow (Fig. 4a–d). Model C is an instance of the SC model while model D is an instance of the IIM model. Also gene flow is between nonsister species in model C and between sister species under model D. We simulate data under models A, B, C, or D and analyze them under models C and D. We refer to our simulation settings in the format of simulation model–analysis model. For example, in the A-C setting, data are simulated under model A and analyzed under model C (Fig. 4). Note that gene flow may be misspecified in two ways. First, the mode of gene flow may be misspecified (A-C and B–D settings). Second, gene flow may be assigned to a wrong branch (C-D and D-C settings). We also consider a combination of both kinds of misspecification (B-C and A-D settings).

The parameter values used (including species split times, introgression time, population size, and introgression probability) are in the legend to Fig. 4. As before each replicate dataset consists of L=250, 1,000, or 4,000 loci, with S=4 sequences per species per locus and with n=500 sites in the sequence.

The posterior means and 95% HPD CIs of parameters are summarized in Fig. 4e (see also supplementary table S1, Supplementary Material online). The true and fitted distributions of coalescent times are in supplementary fig. S8, Supplementary Material online. We also conducted the Bayesian test of gene flow using the same data, with results summarized in supplementary fig. S9, Supplementary Material online. Our discussion below may refer to Huang et al. (2022, Fig. 4), where complementary results from using the MSC-I models to analyze data generated under the MSC-M models (e.g. the C-A, C-B, D-A, D-B settings) can be found.

#### Inference Under the Correct Model (Fig. 4, C-C and D-D)

The two cases where the analysis model matches the simulation model, C-C and D-D (Fig. 4e), represent the best-case scenarios and serve as a reference for comparison. First, we note that the Bayesian test of gene flow has full power in those two settings, even in the small datasets (supplementary fig. S9, Supplementary Material online, C-C and D-D). Second in both settings most parameters including the migration rate are well estimated, with narrow CIs covering the true parameter values (Fig. 4e, supplementary table S1, Supplementary Material online).

Parameters $M,\theta_{S},\tau_{S}$, related to the gene-flow event, have wider CIs in the D-D setting than in the C-C setting. This may be due to two factors. First, it may be harder to estimate the rate of gene flow between sister lineages (D-D) than between nonsister lineages (C-C); for example, migration between nonsisters may cause a change to the gene-tree topology, making it easy for the method to identify migrant sequences. Second, it may be harder to estimate the rate of gene flow involving ancestral species since fewer sequences may reach the time of gene flow when one traces the history of sampled sequences backwards in time. While four sequences are from species *B* in the C-C setting, on average fewer than four sequences from species *B* and *C* reach the time of migration ($\tau_{T}$) in the D-D setting; see, e.g. fig. S1 in Thawornwattana et al. 2023a) for time T=1 or 3 coalescent units and note that here time is 2 coalescent units ($\tau_{T}=\theta_{0}$).

#### Inference When Gene Flow is Assigned to a Wrong Lineage (Fig. 4, C-D and D-C)

In the C-D setting, data are generated under model C with migration occurring after a period of isolation, but analyzed under model D with migration assigned to the wrong lineage of parental species. Parameters for populations far away from the migration event, such as the root divergence time and population size ($\tau_{R},\theta_{R}$) and the outgroup population size ($\theta_{D}$), are well estimated (Fig. 4, C-D). Other parameters have serious biases. Due to migration in the true model C, sequences from *A* are expected to be closer to those from *B* than to those from *C*, with $t_{ab}<t_{ac}$. However, the fitting model D predicts equal distances ($t_{ab}=t_{ac}$). There is a serious mismatch between the true and fitting models in the expected distributions of coalescent times (supplementary fig. S8, Supplementary Material online). Under model D, divergence time $\tau_{S}$ (and thus $\tau_{T}$) are severely underestimated to accommodate the small $t_{ab}$ in the data, with ${\hat{\tau }}_{S}=0.0032$ and ${\hat{\tau }}_{T}=0.0011$ (supplementary table S1, Supplementary Material online, C-D) while the true values are $\tau_{S}=0.006$ and $\tau_{T}=0.002$. With $\tau_{S}$ underestimated, $\theta_{S}$ is overestimated as well. The estimates of $\theta_{B}$ and $\theta_{C}$ are also affected, with ${\hat{\theta }}_{B}$ overestimated and ${\hat{\theta }}_{C}$ underestimated. Another conflict is that in the fitting model D, $t_{ab}$ and $t_{ac}$ have the same distribution while they are different under the true model C (supplementary fig. S8, Supplementary Material online, third row). This leads to a poor fit of $t_{ac}$. Estimates of the migration rate and recipient population size ($\hat{M}$ and ${\hat{\theta }}_{T}$) are unreasonably large. However, their ratio or the mutation-scaled migration rate $M/\theta_{T}=m/\mu$ is much better estimated, with $\hat{M}/{\hat{\theta }}_{T}=90.4,105.9$, and 116.3 for L=250, 1,000 and 4,000, respectively, compared with the true value in model C of $M/\theta_{T}=100$ (supplementary table S1, Supplementary Material online). This suggests that the proportion of migrants (*m*) has a greater impact on the distribution of gene trees and coalescent times than the number of migrants (M=Nm).

In the D-C setting, migration occurs initially after species divergence (i.e. IIM) but the analysis model assumes SC, with gene flow mis-assigned to the wrong daughter lineage. Population sizes of the modern species ($\theta_{A},\theta_{B},\theta_{C},\theta_{D}$) are very well estimated, as are the population size and age of the root ($\theta_{R},\tau_{R}$) and the divergence time $\tau_{T}$ between *B* and *C*. However, $\tau_{S}$ and $\theta_{S}$ are grossly biased (Fig. 4e). The estimated migration rate is very low (${\hat{M}}^{D-C}=0.0011$; supplementary table S1, Supplementary Material online). As a result, there is an excess of coalescent times $t_{ab}$ and $t_{ac}$, and a deficit in $t_{bc}$, over the time interval ($\tau_{T}$, $\tau_{S}$) in the data (caused by coalescent events between *A* and *T* in the true model) (supplementary fig. S8, Supplementary Material online). These are explained in the fitting model D by having a very recent divergence time $\tau_{S}$ between *A* and *T*, which is estimated to be close to ${\hat{\tau }}_{T}^{D-C}$, with ${\hat{\tau }}_{S}^{D-C}=0.0028<\tau_{S}^{D}$ and ${\hat{\tau }}_{T}^{D-C}=0.0020=\tau_{T}^{D}$ (supplementary table S1, Supplementary Material online). Because of this underestimation of $\tau_{S}$, $\theta_{S}$ is overestimated. Overall, the fitted distribution of $t_{bc}$ matches the true distribution under model C reasonably well while the fitted values of $t_{ab}$ and $t_{ac}$ reflect the increased duration (${\hat{\tau }}_{S}$,${\hat{\tau }}_{R}$) of population *S*, with the majority of coalescent events occurring closer to ${\hat{\tau }}_{S}$ (supplementary fig. S8, Supplementary Material online).

Consistent with the large estimates of *M* in the C-D setting, the Bayesian test of gene flow has full power (supplementary fig. S9, Supplementary Material online, C-D). In contrast, in the D-C setting, the estimated migration rate is very low, and the Bayesian test has virtually zero power (supplementary fig. S9, Supplementary Material online, D-C). Note that in both models C and D, continuous migration (MSC-M) is assumed. Huang et al. (2022, Fig. 4) examined the A-B and B-A settings, in which the discrete introgression model (MSC-I) is used. In the A-B setting, large estimates of the introgression probability (φ) are produced, while in the B-A setting, the estimated φ is near zero.

Thus we observe the same patterns regardless of the mode of gene flow (MSC-I or MSC-M). If gene flow is between nonsister species but is mis-assigned to the parental lineage so that the assumed gene flow is between sister species (the A-B and C-D settings), we will obtain large estimates of the rate of gene flow, and the Bayesian test will infer gene flow. In contrast, if gene flow involves an ancestral branch and is between sister species but mis-assigned to a daughter branch (the B-A and D-C settings), we will obtain low estimates of the rate of gene flow and the Bayesian test may not detect gene flow. Nevertheless, in our simulations (Fig. 4 in this study and Fig. 4 in Huang et al. 2022) the impacts of mis-assigning a gene-flow event onto parental or daughter branches are local, mostly affecting parameters for lineages on the species tree involved in gene flow.

#### Inference When the Mode of Gene Flow is Misspecified (Fig. 4, A-C and B-D)

Next, we consider cases where the mode of gene flow is misspecified, with data generated under MSC-I and analyzed under MSC-M, but the population pair involved in gene flow is correctly specified. In the A-C setting, gene flow is between nonsister species while in the B-D setting, it is between sister species. This is an extension of our two-species analysis (Figs. 1–3) to a larger phylogeny. Previously, Huang et al. (2022, Fig. 4) examined the opposite settings, C-A and D-B, in which data were simulated under MSC-M and analyzed under MSC-I, noting that highly precise and accurate parameter estimates were obtained despite the misspecification of the mode of gene flow. In particular, in the case of SC (the C-A setting), the MSC-I model was able to recover almost all gene flow that occurred under the migration model (with $\hat{\varphi }$ under MSC-I being close to $\varphi_{0}$ under MSC-M).

In both the A-C and B-D settings, all population-size and divergence-time parameters are reliably estimated at comparable levels of precision to the C-C and D-D settings. Surprisingly, some parameters in the B-D setting, such as $\tau_{S}$ and $\theta_{S}$, appear to be even more precisely estimated than in the D-D setting (Fig. 4, supplementary table S1, Supplementary Material online). A similar observation was made in the C-A setting in comparison with A-A (Huang et al. 2022). There is a slight overestimation of $\theta_{S}$: ${\hat{\theta }}_{S}^{A-C}=0.003$ and ${\hat{\theta }}_{S}^{B-D}=0.0024$ while the true value is 0.002 (supplementary table S1, Supplementary Material online).

We obtain a larger estimate of the migration rate in the B-D setting than in the A-C setting even though the introgression probability is the same (φ=0.2), with ${\hat{M}}^{B-D}=0.0405$ compared with ${\hat{M}}^{A-C}=0.0116$ (supplementary table S1, Supplementary Material online). A high migration rate (*M*) in model C predicts the existence of very small coalescent times $t_{ab}$ or the existence of nearly identical sequences from *A* and *B*, and is thus incompatible with the data, which is generated under model *A* with gene flow at a fixed time point in the past ($\tau_{X}>0$). This is the same pattern as observed in our analysis of the two-species case, where the IIM model (here, model D) recovers more gene flow and provides less biased parameter estimates than the IM or SC models (Fig. 3).

The Bayesian test of gene flow has ∼100% power in all datasets in the A-C and B-D settings (supplementary table S9, Supplementary Material online). Gene flow is detected despite the misspecification of the mode of gene flow.

In summary, the misspecification of the mode of gene flow does not have large detrimental effects in our simulations (Fig. 4, A-C and B-D, and Huang et al. 2022, Fig. 4, C-A and D-B). If gene flow occurred in a pulse in the past but has since stopped, MSC-M models assuming ongoing gene flow (IM and SC) may underestimate the amount of gene flow, while the IIM model produced more accurate estimates. When gene flow occurs over extended time periods (as assumed in MSC-M), the MSC-I model is able to produce highly reliable parameter estimates and the test has high power for detecting gene flow.

#### Inference When Both the Mode of Gene Flow and the Introgression Lineage are Misspecified (Fig. 4, B-C and A-D)

Lastly, we examine the cases when both the mode of gene flow and the lineages involved in gene flow are misspecified (Fig. 4: B-C, A-D). From the discussions above, we expect the mis-assignment of lineages involved in gene flow to have more impact than the misspecification of the mode of gene flow. The results confirm this expectation.

In the B-C setting, introgression occurs from *A* to *T* at $\tau_{X}$ prior to the divergence of *B* and *C* at $\tau_{T}$ while the fitting model assumes continuous gene flow from *A* to *B*. As model C assumes ongoing gene flow, which is absent in the data, the estimated migration rate *M* is close to zero ($\hat{M}<10^{-3}$; supplementary table S1, Supplementary Material online), and the Bayesian test has zero power in detecting gene flow in any of the datasets (supplementary fig. S9, Supplementary Material online). The result is very similar to the D-C setting. Apart from the serious underestimation of the rate of gene flow (*M*), other effects of model misspecification are local, affecting mainly $\tau_{S}$ and $\theta_{S}$. All modern population sizes ($\theta_{A}$, $\theta_{B}$, $\theta_{C}$, and $\theta_{D}$) as well as $\tau_{R}$ and $\theta_{R}$ are well estimated. Model C can fit coalescent times during the initial period $\left(0,\tau_{T}\right)$ well without requiring gene flow. Small coalescent times $t_{ab}$ and $t_{ac}$ due to gene flow in the data generated under model B are then explained by having a more recent divergence time $\tau_{S}$. We obtain ${\hat{\tau }}_{S}=0.0039$, which is much closer to the introgression time $\tau_{X}=0.003$ than the true divergence time $\tau_{S}=0.006$. The root divergence time and population size are slightly affected, with $\tau_{R}$ slightly overestimated and $\theta_{R}$ slightly underestimated.

In the A-D setting, introgression occurs from *A* to *B*, a nonsister species, while the fitting model assumes continuous gene flow from *A* to its sister lineage *T*. As in the C-D setting, the gene-flow event is mis-assigned onto the parental branch, leading to large estimates of the migration rate (*M*) and the Bayesian test has full power (supplementary fig. S9, Supplementary Material online). As before, the impacts on other parameters are largely local. All present-day population sizes ($\theta_{A},\theta_{B},\theta_{C},and\theta_{D}$), $\tau_{R}$ and $\theta_{R}$ are well estimated.

### Inference When the Direction of Gene Flow is Misspecified on a Four-species Phylogeny

**Fig. 5.**
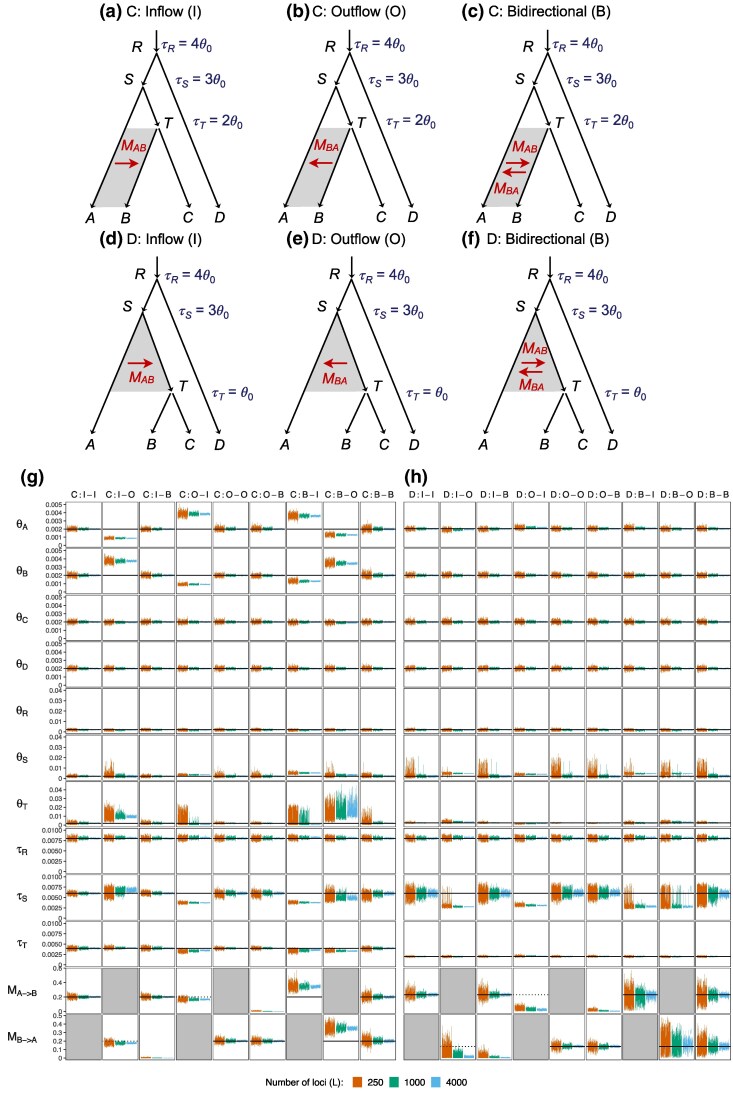
a–f) MSC-M models with different directions of gene flow between nonsister lineages *A* and *B* (a–c, model C) or between sister lineages *A* and *T* (d–f, model D), with either inflow (b and d), outflow (c and e), or bidirectional gene flow (d and f). The notation C:I-O means the data were generated under model C:I (inflow) and analyzed under model C:O (outflow), etc. Parameters used to generate the data are the same as those in Fig. 4c and d. All migration rates were 0.2. For D models, the migration rate $M_{A\to T}$ is labeled $M_{A\to B}$, etc. for convenience. g–h) The 95% HPD CIs for parameters in 100 replicate datasets of L=250,1,000, and 4,000 loci for C models (g) and D models (h). Black solid line indicates the true value. Estimates for the C:I-I and D:I-I settings are identical to those for the C-C and D-D settings, respectively, in Fig. 4. A gray box indicates that the parameter does not exist in the model.

Previously, we studied the effects of misspecified direction of gene flow on parameter estimation and the Bayesian test of gene flow under the MSC-I model (Thawornwattana et al. 2023a). Here, we perform complementary analysis under the MSC-M model. We consider the two MSC-M models of Fig. 4c and d: C (recent gene flow involving nonsister species) and D (ancestral gene flow involving sister species), and, for each, consider three variants: inflow (I, A→B), outflow (O, B→A), and bidirectional gene flow (B, A⇆B), as shown in Fig. 5a–f. Models I, O, and B make different assumptions about the direction of gene flow while both the lineages involved and the mode of gene flow (continuous migration) are correctly specified. For example, in the C:I-O setting (Fig. 5g), data are generated under the inflow model with A→B migration (Fig. 5a) but analyzed under the outflow model assuming B→A migration (Fig. 5b), so that the assumed direction is the opposite. The I-B and O-B settings represent over-parametrization rather than misspecification.

We used the same parameter values as in the previous section (Fig. 4c–d). Results are summarized in Fig. 5g and h. Estimates from the large datasets of L=4,000 loci under models C and D are in supplementary tables S2 and S3, Supplementary Material online, respectively.

#### Analysis Under the SC Model (Model C, Fig. 5g)

In model C, gene flow is recent and between nonsister species *A* and *B*. It may be considered an instance of an SC model in which gene flow occurs after a period of complete isolation. As a baseline for comparison, we first consider cases where the analysis model is correctly specified, i.e. C:I-I, C:O-O, and C:B-B (Fig. 5g). In all three cases, all parameters including the migration rates are correctly estimated. The present-day population sizes ($\theta_{A},\theta_{B},\theta_{C},and\theta_{D}$) are estimated with narrow CIs, while there is more uncertainty in the ancestral population sizes ($\theta_{T},\theta_{S},and\theta_{R}$) (Fig. 5g). We find that the rate of inflow and outflow is estimated at similar levels of precision. We expect the rates of bidirectional gene flow to involve more uncertainties than the unidirectional rates and this is indeed the case for datasets of L=250 or 1,000 loci (see *M* estimates under the I-I, O-O, and B-B settings in Fig. 5g), but the CIs have the same widths in large datasets of L=4,000 loci (see estimates of *M* in supplementary table S2, Supplementary Material online, model C: I-I, O-O, and B-B). The Bayesian test of gene flow has full power in all datasets (supplementary fig. S10a–c, Supplementary Material online for C:I-I, C:O-O, and C:B-B).

In the other settings (I-O, O-I, B-I, and B-O; Fig. 5g), the direction of gene flow is misspecified although the I-B and O-B settings represent over-parametrization rather than misspecification. Overall, the impact of misspecification on species divergence times and population sizes is local, affecting parameters for populations involved in gene flow or their immediate ancestors. For example, the population size and divergence time at the root of the tree ($\tau_{R}$ and $\theta_{R}$) are well estimated. Below we focus on parameters that are affected by the misspecified direction of gene flow, in particular, the migration rates.

In the C:I-B and C:O-B settings, migration is unidirectional but the analysis model allows for migration in both directions. We recover the correct migration rate in the correct direction while the migration rate in the opposite direction is estimated to be zero (Fig. 5g, supplementary table S2, Supplementary Material online). The CI widths of all parameters are comparable with those obtained from the unidirectional migration case (compare C:I-B with C:I-I and C:O-B with C:O-O), suggesting that overparameterization has a minor impact on parameter estimates. The cost of including a nonexistent migration rate in the bidirectional model (B) is thus mostly computational. The Bayesian test of gene flow supported the true migration rate with 100% power, and rejected the nonexistent gene flow with a false positive rate of 0% (supplementary fig. S10a–b, Supplementary Material online for C:I-B and C:O-B).

In the C:I-O and C:O-I settings, migration occurs in one direction but the analysis model assumes the opposite direction. The estimated migration rate in the wrong direction is not zero or negative, but is comparable with the true rate in the opposite direction. For example, in the C:I-O setting the estimate is ${\hat{M}}_{B\to A}=0.176$ with the 95% CI to be (0.168, 0.184) (supplementary table S2, Supplementary Material online, C:I-O), compared with $M_{A\to B}=0.2$ in the true model. Furthermore, the Bayesian test detected gene flow in all datasets (supplementary fig. S10a–b, Supplementary Material online for C:I-O and C:O-I). This may be considered as a false positive rate if one emphasizes the inferred wrong direction or power if one emphasizes the presence of gene flow.

Misspecification of the direction of gene flow has a local effect on estimated population-size parameters and divergence times for populations involved in gene flow (A,B,T, and the ancestor *S*) (Fig. 5g, C:I-O and O-I). When the true recipient population is incorrectly assumed to be a source population, we expect its population size to be overestimated to account for the excess polymorphism. Conversely, a source population incorrectly assumed as a recipient should have its population size underestimated. The results confirm those expectations (Fig. 5g, supplementary table S2, Supplementary Material online). For example, in the C:I-O setting, gene flow is from A→B but assumed to be from B→A. Thus $\theta_{A}$ is grossly underestimated and $\theta_{B}$ overestimated. The large biases in population sizes may also be accompanied by biases in species divergence times ($\tau_{T},\tau_{S}$) (Fig. 5g, C:I-O and O-I) when the model attempts to fit the coalescent times for sequences from the same species ($t_{aa},t_{bb}$).

In the C:B-I and C:B-O settings, migration occurs in both directions while the analysis model incorrectly assumes unidirectional migration. Gene flow in both directions does not cancel out (unlike debts and credits), and instead shows a cumulative effect. The estimate migration rates are 0.34 and 0.35 in the C:B-I and C:B-O settings, much higher than 0.17 and 0.18 in the O-I and I-O settings, respectively. This may not be so surprising when one considers that gene flow in either direction reduces sequence divergence between the two species involved. Similarly, the Bayesian test detects gene flow with full power in all datasets for those settings (supplementary fig. S10c, Supplementary Material online, C:B-I and C:B-O). The effects on the estimation of population sizes and species divergence times are similar to the C:O-I and C:I-O settings. For example, the population size for the incorrectly assumed source population (*A* in C:B-I and *B* in C:B-O) is overestimated and that for the assumed recipient population (*B* in C:B-I and *A* in C:B-O) is underestimated (Fig. 5g). The cumulative effect of gene flow in both directions in the true model tend to reduce the coalescent time $t_{ab}$ between sequences from species involved in gene flow (*A* and *B*), and consequently, $\tau_{T}$ and $\tau_{S}$ are underestimated (supplementary table S3, Supplementary Material online).

#### Analysis Under the IIM Model (Model D, Fig. 5h)

Lastly, we consider model D, which has ancestral gene flow between sister lineages *A* and *T* before *T* splits into two species *B* and *C* (Fig. 5d–f). This may be considered as an instance of an IIM model (Fig. 1c).

First, we note that in all settings for model D, population sizes for extant species are well estimated (Fig. 5h), because there is no gene flow during the time period ($\tau_{T},0$) in either the true or the analysis models. This is different from the settings based on model C, in which extant species may be the source or donor populations of gene flow. Divergence time $\tau_{T}$ and population size $\theta_{T}$ are also well estimated in all settings for model D.

When the model is correctly specified (D:I-I, D:O-O, and D:B-B), the Bayesian test of gene flow has ∼100% power, even in small datasets with 250 loci (supplementary fig. S10d–f, Supplementary Material online). All parameters are well estimated, with the CIs becoming narrower with the increase of the data size (the number of loci). Again population sizes for ancestral species ($\theta_{S},\theta_{T}$) have far wider CIs than those for modern species. Migration rates ($M_{A\to B}$ and $M_{B\to A}$) as well as $\tau_{S}$ and $\theta_{S}$ have wider CIs under the D models (Fig. 5h) than under the corresponding C models (Fig. 5g). Inference of gene flow under model D is more challenging than under model C. This may be due to two factors. First, gene flow in D is more ancient. Second, gene flow in D is between sister lineages while that in C is between nonsisters. See our discussion above of the C-C versus D-D settings in Fig. 4e (which correspond to the C:I-I and D:I-I settings in Fig. 5g and h).

In the D:I-B and D:O-B settings, the analysis model assumes bidirectional migration while migration is in fact unidirectional. The results under D:I-B are very similar to those under D:I-I (and D:O-B to D:O-O), while the rate of migration that does not exist in the true model estimated to be zero (Fig. 5h, supplementary table S3, Supplementary Material online). The over-parametrization of the B model has no major impact on the estimation. The Bayesian test detected gene flow in the correct direction with full power while rejecting the nonexistent gene flow in the opposite direction with false positive rate of ∼0% (supplementary fig. S10d and e, Supplementary Material online, D:I-B and D:O-B).

In the D:I-O and D:O-I settings, gene flow is unidirectional but the assumed direction is the opposite. Migration rate is estimated to be close to zero, and the divergence time $\tau_{S}$ is seriously underestimated, as the number of loci increases (Fig. 5h, supplementary table S3, Supplementary Material online). Furthermore, the Bayesian test often fails to detect gene flow (supplementary fig. S10d–e, Supplementary Material online). Apparently, misspecification of migration direction caused the method to misinterpret early divergence with gene flow as recent complete isolation with no gene flow. This is in contrast to the C:I-O and C:O-I settings where the estimated migration rate between nonsister species in the wrong direction is positive and close to the true value (Fig. 5g). It is also in contrast to the C:I-I and C:O-O settings where there is no model misspecification and the method is able to distinguish between early divergence with gene flow and recent complete isolation with no gene flow.

In the D:B-I and D:B-O settings, migration occurs in both directions but the analysis model allows only one direction. Migration rate in the allowed direction is estimated to be around the true rate in that direction, with wide CIs (even wider than in the D:B-B setting) (Fig. 5h, D:B-I and D:B-O). Here gene flow in the two directions does not show a cumulative effect, in contrast to the C:B-I and C:B-O settings discussed above. Consistently with parameter estimation, the Bayesian test of gene flow has only moderate power in the D:B-I and D:B-O settings, in contrast to the full power in the C:B-I and C:B-O settings (supplementary fig. S10f vs. fig. S10d, Supplementary Material online). The model underestimates the amount of gene flow between species *A* and *T*, and this is compensated by an underestimation of their split time ($\tau_{S}$; Fig. 5g, D:B-I and D:B-O).

In summary, when the direction of gene flow is correctly specified, the Bayesian test has high power to detect gene flow both between sister lineages and between nonsister lineages. While ancient gene flow between sister lineages is harder to infer than recent gene flow between nonsister species, the Bayesian test easily achieves full power in both scenarios (supplementary fig. S9, Supplementary Material online, C-D and D-D), and the precision in the estimated rate of gene flow is comparable (cf: C:I-I with D:I-I, and C:O-O with D:O-O in Fig. 5g and h). When the direction of gene flow is misspecified, one can easily infer gene flow between extant nonsister lineages (with a cumulative effect if gene flow occurs in both directions). However, misspecification of the direction of gene flow between ancestral sister lineages makes it difficult to detect gene flow.

### Analysis of Data from Purple Cone Spruce

**Fig. 6.**
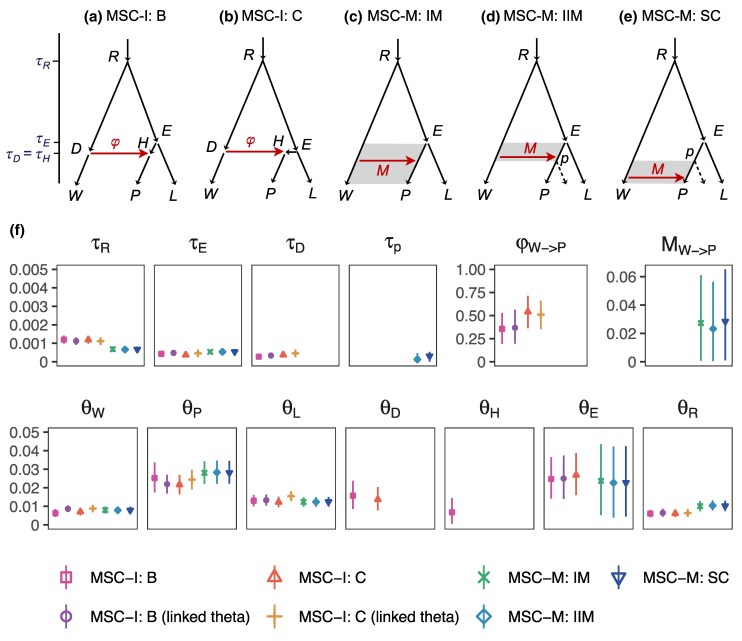
a–b) Two MSC-I models (B and C; Flouri et al. 2020) and c–e) three MSC-M models (IM, IIM, and SC; Huang et al. 2022; Flouri et al. 2023) for three purple cone spruce species: *Picea wilsonii* (W), *P. purpurea* (P), and *P. likiangensis* (L). f) Posterior means and 95% HPD CIs for parameters in the models obtained in Bpp analysis of genomic data. MSC-I modes B and C are implemented either with and without the linked-theta option, which forces *θ* to be the same before and after introgression.

To gain insights into Bayesian parameter estimation and the Bayesian test of gene flow when the true history of species divergence and gene flow is unknown, we analyzed an empirical dataset from three purple cone spruce species, *Picea wilsonii* (W), *P. likiangensis* (L), and *P. purpurea* (P) (Sun et al. 2014 ). The purple cone spruce is endemic to the Qinghai-Tibet Plateau, and *P. purpurea* is hypothesized to be a hybrid species, formed through homoploid hybridization between *P. wilsonii* and *P. likiangensis* (Sun et al. 2014). Thus, the MSC-I model assuming a pulse of hybridization/introgression may be expected to be a better fit to the data than the MSC-M model. We considered two variants of the MSC-I model (Fig. 6a–b) and three variants of the MSC-M model: IM, IIM, and SC (Fig. 6c–e). Results of Bayesian model comparison are summarized in supplementary table S4, Supplementary Material online, while parameter estimates under those models are in supplementary table S5, Supplementary Material online and Fig. 6f.

Both MSC-I models B and C support the hypothesis that *P. purpurea* is an admixture or hybrid between *P. wilsonii* and *P. likiangensis*. Estimates of the introgression probability (contribution of the *P. wilsonii* parent) range over 0.35 to 0.54, close to 50% (supplementary table S5, Supplementary Material online, Fig. 6f). Estimated divergence and hybridization times are much smaller than the average coalescent time between two sequences sampled within the same species (θ/2), indicating the very recent nature of those species.

Bayesian model comparison strongly favors the MSC-I models over the MSC-M models (supplementary table S4, Supplementary Material online), consistent with the hybrid origin of *P. purpurea*. We further test whether gene flow from the two parental lineages into *P. purpurea* occurred at the same time, as predicted by the hypothesis of hybrid speciation. The null hypothesis is model C with the constraint $\tau_{D}=\tau_{E}$ (Fig. 6b), while the alternative hypothesis is model B with $\tau_{D}<\tau_{E}$ (Fig. 6a). This test is inclusive, as the Bayes factor is in the range (0.01, 100) and does not strongly favor either model (supplementary table S4, Supplementary Material online).

Given that the MSC-I models fit the data better, we next examine biases in parameter estimates that result from the use of “wrong” MSC-M models. All three migration models (IM, IIM, and SC; Fig. 6c–e) produced lower rates of gene flow and more recent divergence time ($\tau_{R}$). The estimated migration rate *M* correspond to $\varphi_{0}<1\%$, much lower than estimates of φ under the MSC-I models (Fig. 6f; supplementary table S5, Supplementary Material online). In effect, the MSC-M models mis-interpreted early divergence with gene flow as recent divergence with very little gene flow.

This is the same pattern found in our simulations, in which the IM model may misinterpret early divergence with gene flow as more recent divergence with no or little gene flow (Fig. 2 for *M* in the case of two species and Fig. 4e, A-C in the case of four species). Overall, the analysis of the empirical data showed the same patterns as found in the asymptotic analysis and computer simulation. As expected from the hypothesis of hybrid speciation (Sun et al. 2014), our Bayesian model test strongly favors the pulse model of gene flow over continuous migration.

## Conclusions

Here we summarize our key findings from this simulation study, by integrating with the results from previous studies, which used the MSC-I model for data analysis (Jiao et al. 2020; Huang et al. 2022; Ji et al. 2023; Thawornwattana et al. 2023a).

First, the Bayesian test of gene flow has high power in detecting both recent and ancient gene flow, either between nonsister species or between sister lineages. In previous simulations, the Bayesian test was found to have much higher power than summary methods based on genome-wide site pattern counts such as Hyde (Ji et al. 2023, Fig. 9) or on gene-tree counts such as Snaq (Ji et al. 2025, Fig. 5).

When the mode of gene flow is misspecified (i.e. in the I-M and M-I settings), the Bayesian test is found to have high power in almost all cases we have examined. We generated data under the MSC-I model and analyzed them using variants of the MSC-M model (IM, IIM, and SC). Despite the misspecification of the mode of gene flow, the test detects gene flow in most cases, whether gene flow involved sister lineages (supplementary fig. S7, Supplementary Material online) or nonsister lineages (supplementary fig. S9 A-C and B-D and fig. S10, Supplementary Material online). This means that one is very likely to infer gene flow even if the assumed mode of gene flow is not a perfect match to reality (for example, if the rate of gene flow varies over time). We observed low power in the case of two species when φ is very low or very high (supplementary fig. S7, Supplementary Material online, φ), in which case the MSC-I model is close to the MSC model with no gene flow.

Second, misspecification of the mode of gene flow leads to underestimation of the amount of gene flow. In other words, in both the I-M and M-I settings, Bayesian estimation tends to recover less gene flow than in the true model, as measured by the total amount of gene flow as a fraction of the expected number of immigrants in the recipient population (that is, φ in MSC-I or $\varphi_{0}$ in MSC-M) (e.g. Fig. 3 and Fig. 4e, A-C). When the true model is MSC-I, the IM and SC models in particular produce serious underestimates of the amount of gene flow, because those models assume ongoing gene flow up to the present time and predict recent coalescent times between sequences from the two species (with $t_{ab}$ near zero), which do not exist in the data. In such cases, the IIM model recovers a greater amount of gene flow (Fig. 3). Previously, the discrete model (MSC-I) was found to recover less than the true amount of gene flow when gene flow occurs over extended time periods according to the MSC-M model (Huang et al. 2022, Fig. 1e).

Third, while it is harder to infer gene flow between sister lineages than between nonsisters, with multiple sequences sampled per species, the Bayesian method is powerful in inferring gene flow between sister lineages: the Bayesian test based on the Bayes factor has high power to detect gene flow and Bayesian estimation produces estimates of the rate of gene flow with precision similar to the case of nonsister gene flow (e.g. Fig. 4e, D-D for the MSC-M model; see also Fig. 4, B-B in Huang et al. 2022 for the MSC-I model). Note that most summary methods (such as the *D*-statistic and the *f*-branch test) are unable to identify gene flow between sister lineages. For triplet methods based on gene trees, introgression between nonsister lineages causes changes to the gene tree topology, whereas introgression between sister lineages does not. For triplet methods based on site-pattern counts, introgression between nonsister lineages causes an asymmetry in the site-pattern counts, but introgression between sister lineages does not.

Fourth, when the direction of gene flow is correctly specified, Bayesian test has high power to detect gene flow both between sister lineages and between nonsister lineages. While ancient gene flow between sister lineages is harder to infer than recent gene flow between nonsister species, the Bayesian method can achieve similar power in the test of gene flow and similar precision in the estimated rate of gene flow (cf: C:I-I with D:I-I, and C:O-O with D:O-O in Fig. 5g and h). When the direction of gene flow is misspecified, the Bayesian test often detects gene flow. The estimated rate of gene flow may be higher or lower than the true rate in the opposite direction (e.g. Fig. 5g and h, I-O, O-I for the MSC-M model and Fig. 4 and supplementary fig. S4, Supplementary Material online in Thawornwattana et al. 2023a for MSC-I). When gene flow occurs in both directions but a unidirectional model is assumed, gene flow in the two directions does not cancel out and may instead show a cumulative effect. If gene flow is unidirectional, use of the bidirectional model leads to detection of gene flow in the correct direction, and rejection of gene flow in the wrong direction (supplementary fig. S10, Supplementary Material online, C:I-B, C:O-B, D:I-B, and D:O-B; see also (Thawornwattana et al. 2023a), supplementary fig. S2, Supplementary Material online, model B). Unlike Frequentist hypothesis testing, the Bayesian test may lead to strong rejection of the more general alternative hypothesis. Note that most summary methods cannot identify the direction of gene flow (Jiao et al. 2021; Huang et al. 2022).

Fifth, when the gene-flow event is mis-assigned to a parental or daughter branch rather than the lineage genuinely involved in gene flow, the Bayesian method may produce highly biased parameter estimates (Fig. 4e, C-D and D-C), and the Bayesian test may fail to detect the gene flow (supplementary fig. S10, Supplementary Material online, D-C). Under the MSC-I model, the inferred introgression time tends to be stuck on the species divergence time, and the estimated rate of gene flow tends to be far lower than the true rate (Huang et al. 2022, Fig. 4e, A-B and B-A).

Finally, misspecification of the mode of gene flow (MSC-I versus MSC-M) tends to have only small local effects, affecting divergence times and population sizes for species on the phylogeny around the lineages involved in the gene flow (e.g. Fig. 4e, A-C and B-D for MSC-M). The Bayesian test has high power despite the misspecification; for example, if the true model is MSC-I but data are analyzed under MSC-M, the test may still have full power (supplementary fig. S9, Supplementary Material online, A-C and B-D).

Overall, analysis of both synthetic and real datasets in this and previous studies demonstrate that the MSC-I and MSC-M models, no doubt extreme simplifications of gene flow in the real world, are very effective for detecting gene flow and estimating its rate using genomic sequence data. The MSC-I model in particular performed well in simulations under MSC-M with gene flow over extended time periods. The MSC-M models may be most suitable to data from different populations of the same species where gene flow may be ongoing and over extended time periods. Variants of the model such as IM, IIM, and SC make different assumptions about possible gene flow following speciation, and may be useful for testing different theories of speciation (Westram et al. 2022).

## Materials and Methods

### Two Species Case: Asymptotic Analysis

The case of two species when the data consist of one sequence per species is simple enough to yield analytical solutions. We considered estimation of parameters under the three MSC-M models of Fig. 1b–d when data of an infinite number of loci (L→∞) were generated under the MSC-I model of Fig. 1a. We obtained the limit of the MLEs, $\theta_{m}^{*}$, by minimizing the KL divergence (equation (3)). As L→∞, the data are represented by the distribution of the number of differences between two sequences at the *n* sites, and maximizing the likelihood is equivalent to minimizing the KL divergence. Optimization was achieved using a C program that implements the BFGS algorithm from Paml (Yang 2007). The program is available at https://github.com/ythaworn/iimmsci2s. For each model and each value of φ, we ran the optimization multiple times and used the run with the lowest KL value. We excluded runs with parameter values on the optimization boundaries.

### Two Species Case: Simulation

We used simulation to verify and extend our asymptotic analysis. We simulated multilocus sequence data under the MSC-I model of Fig. 1a and analyzed them under the IM, IIM, and SC models of Fig. 1b–d. We used two values of population sizes on the species tree: $\theta_{A}=\theta_{X}=\theta_{R}=\theta_{0}=0.002$ (thin branches) and $\theta_{B}=\theta_{Y}=\theta_{1}=0.01$ (thick branches). Introgression occurred from species *A* to *B* with probability φ=0.2 at time $\tau_{X}=\theta_{0}$ after species divergence at time $\tau_{R}=2\theta_{0}$. In the base case, we assumed φ=0.2 and the data consisted of L=4,000 loci, with S=4 sequences per species, and n=1,000 sites per sequence. We varied the following four factors one at a time, keeping other parameters fixed: the number of sites per sequence (*n*), the number of sequences per species (*S*), the number of loci (*L*), and the introgression probability (φ). The values used were n=250, 1,000, 4,000, 16,000, 64,000; S=1,2,4,8,16; L=250, 500, 1,000, 2,000, 4,000, 8,000; and φ=0.01, 0.05, 0.1, 0.2, 0.3, 0.4, 0.5, and 0.7. For each setting, we simulated 30 replicate datasets. With those four factors (n,S,L,M), and 30 replicates each, there were (5+5+6+8−3)×30=630 datasets in total. The subtraction by 3 accounted for the fact that all four factors shared the same base case (n=1,000, S=4, L=4,000, φ=0.2). To generate sequence data, we first generated a gene tree with coalescent times at each locus and then simulated sequences along branches of the gene tree under the JC model (Jukes and Cantor 1969). Sequences at the tips of the tree became data at the locus. Simulation was done using the simulate option in Bpp v4.7.0 (Flouri et al. 2018, 2020, 2023).

Each dataset was analyzed under the IM, IIM, and SC models of Fig. 1b–d to estimate parameters using Bpp v4.7.0 (Flouri et al. 2023). The JC mutation model was assumed. We assigned gamma priors to population size parameters (*θ*), the root age ($\tau_{R}$) on the species tree and the migration rate: θ∼G(2,200) with mean 2/200=0.01, $\tau_{R}∼G(4,200)$ with mean 4/200=0.02, and M∼G(2,10) with mean 2/10=0.2. For each fitting model, we performed two independent runs of MCMC, each with 32,000 iterations of burnin and $10^{6}$ iterations of the main chain. Samples were recorded every 100 iterations. With three fitting models and two MCMC runs per dataset, there were 3×2×630=3,780 MCMC runs in total. Each run of the base case took about 80 h and 2G of memory while the most expensive runs (L=8,000 or S=8) took about 200 h and 4G of memory. For datasets with S=16, we allowed the MCMC run for up to 300 h and 8G of memory, which was about $4\times 10^{5}$ iterations.

### Four Species Case: Wrong Migration Branch and Wrong Mode of Gene Flow

Data were simulated under the four models (A-D) in a phylogeny for four species in Fig. 4 and analyzed under models C and D. Models A and B assumes discrete introgression, while models C and D assumes continuous migration. Gene flow occurred either between nonsister species (models A and C) or between sister species (models B and D). Parameters are shown in Fig. 4. All population sizes were assumed to be $\theta_{0}=0.002$. For models A and B, we used the introgression probability φ=0.2. For models C and D, we assumed the migration rate M=0.2 migrants per generation. Each dataset consisted of S=4 sequences per species per locus, each of length n=500 sites. We varied the number of loci: L=250, 1,000, and 4,000. For each setting, we simulated 100 replicate datasets.

Each dataset was analyzed under both models C and D (Fig. 4c and d) using Bpp v4.7.0 (Flouri et al. 2023). There were eight settings in total: A-C, B-C, C-C, D-C, A-D, B-D, C-D, and D-D. Settings C-C and D-D served as references for comparison as the model was correctly specified. In settings C-D and D-C, the population pair (or branches in the phylogeny) involved in migration was misspecified. In settings A-C and B-D, gene flow occurred as pulse introgression but was misspecified as continuous migration. Finally, in settings B-C and A-D an incorrect mode of gene flow was assigned to a wrong branch. Gamma priors were assigned to population sizes, root age, and migration rate as θ∼G(2,200) with mean 0.01, $\tau_{R}∼G(4,200)$ with mean 0.02, and M∼G(2,10) with mean 0.2. With four data-generating models, three values of *L* and 100 replicates, there were 4×3×100=1,200 datasets in total. We performed two independent runs of MCMC, each with 10,000 iterations of burnin and $10^{6}$ iterations of the main chain. Samples were recorded every 100 iterations. With two fitting models (C and D) for each dataset, there were 2×2×1,200=4,800 MCMC runs in total. The running time was about 20–30 h for datasets with L=250, 80 h for L=1,000, and 260 h for L=4,000.

### Simulation in the Four Species Case: Misspecified Direction of Gene Flow

To study the effect of incorrectly assumed direction of gene flow under the MSC-M model, we simulated sequence data using models C and D of Fig. 4, but with three specifications concerning the direction of gene flow: inflow (I, A→B), outflow (O, B→A), and bidirectional gene flow (B, A⇆B) (Fig. 5a–f). We used the same parameter values as before (Fig. 4c–d). Each simulated dataset was analyzed assuming the three variants of the MSC-I model (I, O, and B), generating nine settings for model C (e.g. C:I-O) and nine settings for model D (e.g. D:I-O).

We used three values of for the number of loci: L=250, 1,000, and 4,000. For model C, with 3 data-generating models, three values of *L* and 100 replicates, there were 3×3×100=900 datasets in total. MCMC setup was the same as before. With three fitting models (I, O, and B), two independent MCMC runs per dataset, the total number of MCMC runs was 3×2×900=5,400. Similarly for model D, there were 900 datasets and 5,400 MCMC runs in total. We reused the datasets for C:I and D:I from the previous section.

### Bayesian Test of Gene Flow

We conducted the Bayesian test of gene flow (Ji et al. 2023) to assess whether there is significant evidence in the data for gene flow. For example, for unidirectional gene flow (Figs. 1 and 4), the null hypothesis of no gene flow, $H_{0}\;:\;M_{A\to B}=0$ may be compared with the alternative hypothesis of gene flow, $H_{1}\;:\;M_{A\to B}>0$, via the Bayes factor $B_{10}$. As the two hypotheses are nested, $B_{10}$ may be approximated by the Savage–Dickey density ratio, approximated by $B_{10,\varepsilon }=\frac{P(\emptyset )}{P(\emptyset \;|\;X)}$, where P(∅) is the probability for the null interval, $\emptyset \;:\;0<M_{A\to B}<\varepsilon$, under the prior distribution, and P(∅|X) is the corresponding posterior probability. The null interval is part of the parameter space for $H_{1}$ that represents the null hypothesis. We used ε=0.01 and 0.001. $B_{10,\varepsilon }$ was calculated by processing a posterior MCMC sample under $H_{1}$ (Ji et al. 2023). $B_{10}>100$ is considered strong evidence in favor of $H_{1}$, similar to the 1% significance level in hypothesis testing. The power of the test is defined as the proportion of replicate datasets in which $B_{10}>100$.

### Analysis of the Purple Cone Spruce Data

We analyzed a dataset for three purple cone spruce species (*Picea wilsonii*, *P. likiangensis*, and *P. purpurea*) (Sun et al. 2014). There are 11 short nuclear loci (200–600 bp per locus), with 100 sequences from *P. wilsonii*, 112 from *P. purpurea* and 120 from *P. likiangensis*. This is the “full” dataset analyzed by Flouri et al. (2020) under the MSC-I models. Here we used MSC-I and MSC-M models of Fig. 6a–e. We assigned the priors θ∼G(2,200) with mean 0.01, $\tau_{R}∼G(2,1,000)$ with mean 0.002, φ∼U(0,1) for the MSC-I models and w=4M/θ∼G(2,1) with mean 2 for the MSC-M models. For each model, we performed four independent runs of MCMC, each with 40,000 iterations of burn-in and $10^{6}$ main iterations, sampling every 100th iteration. MCMC samples from the four replicate runs were compared to verify convergence before they were pooled to produce final posterior summaries (Fig. 6, supplementary table S5, Supplementary Material online). Each run took ∼60 h for the MSC-I models and 160 h for the MSC-M models.

The Savage–Dickey approach to calculating the Bayes factor applies if the two compared models are nested (as in the test of gene flow). To compare nonnested models (such as the MSC-I and MSC-M models of Fig. 6), we used thermodynamic integration to calculate the marginal likelihood, using 32 Gaussian quadrature points (Lartillot and Philippe 2006; Rannala and Yang 2017) (supplementary table S4, Supplementary Material online). This involved 32 MCMC runs, with the same setup as above. We calculated adjusted Bayes factors by performing least-squares fitting of local quadratic polynomials to stabilize the estimates as described in Thawornwattana et al. (2023b). For comparison of the two variants of the MSC-I model (Fig. 6a and b), which are nested models, we also calculated Bayes factors using the Savage–Dickey density ratio (Ji et al. 2023). We tested whether the introgression time $\tau_{D}$ is significantly different from the species divergence time $\tau_{E}$, with $H_{0}\;:\;\tau_{D}=\tau_{E}$ versus $H_{1}\;:\;\tau_{D}<\tau_{E}$. Bayes factors below 0.01 provide support for $H_{0}$, i.e. hybrid speciation (MSC-I: C; Fig. 6b) while values above 100 support the MSC-I: B model of introgression after divergence (Fig. 6a). Here, we calculated $B_{\varepsilon }=\frac{P(\emptyset )}{P(\emptyset \;|\;X)}$, where P(∅) is the probability of the null interval $\emptyset \;:\;0<\tau_{E}-\tau_{D}<\varepsilon$ under the prior distribution and P(∅|X) is the corresponding probability under the posterior distribution. We used $\varepsilon =10^{-4}$ and $10^{-5}$. We calculated $B_{\varepsilon }$ by processing the MCMC sample for the posterior distribution under $H_{1}$ from the MSC-I: B model (Fig. 6a). The prior null probability P(∅) was calculated from the prior distribution of $\tau_{E}-\tau_{D}$ obtained from running the MCMC without data.

## Supplementary Material

msaf121_Supplementary_Data

## Data Availability

Simulated datasets are available in Zenodo at https://doi.org/10.5281/zenodo.11182437.
