# Supplementary material for: Inference of Gene Flow between Species from Genomic Data When the Mode, Direction, and Lineages are Misspecified
Source: Mol Biol Evol. 2025 Jun 27;42(6):msaf121. doi: 10.1093/molbev/msaf121 (PMC12203007; doi:10.1093/molbev/msaf121)
Supplement: msaf121_Supplementary_Data [file msaf121_supplementary_data.zip › Thawornwattana-2025MBE-SI.pdf]

Supplemental Information
Inference of continuous gene flow between species under misspecified models
Thawornwattana, Y., Flouri, T., Mallet, J., and Yang Z.

SI text: Coalescent time distributions under the MSC-I and MSC-M models for two species

We derive probability densities of coalescent times between two sequences under the MSC-I and MSC-M models for two species of figure 1**a**-**d**. Our asymptotic analysis is based on $f(t_{ab})$, the distribution of coalescent time for two sequences sampled from the two species. We also derive $f(t_{aa})$ and $f(t_{bb})$ for two sequences sampled from the same species, as comparison of the true and fitting distributions of those coalescent times provides important insights into Bayesian parameter estimation under misspecified models (e.g. fig. S6).

Density of coalescent time under the MSC-I model of figure 1a

In the MSC-I model of figure 1**a**, introgression occurs from species A into B at time τ_X with probability φ. With at least two sequences sampled per species, there are eight parameters in total: $\boldsymbol{\theta}_{\text{i}} = \{\varphi, \tau_X, \tau_R, \theta_A, \theta_B, \theta_X, \theta_Y, \theta_R\}$. However, some parameters may be unidentifiable if only one sequence is sampled per species (Yang and Flouri, 2022).

The coalescent time between one sequence from A and one from B has density (e.g., Jiao *et al.*, 2020)

$$f_{\text{i},ab}(t) = \begin{cases} \varphi \frac{2}{\theta_X} \mathrm{e}^{-\frac{2}{\theta_X}(t-\tau_X)}, & \text{if } \tau_X < t \le \tau_R, \\ \left[\varphi \, \mathrm{e}^{-\frac{2}{\theta_X}(\tau_R-\tau_X)} + (1-\varphi)\right] \frac{2}{\theta_R} \mathrm{e}^{-\frac{2}{\theta_R}(t-\tau_R)}, & \text{if } t > \tau_R. \end{cases} \quad \text{(S1)}$$

This is independent of θ_A, θ_B and θ_Y.

For two sequences from A, the density of coalescent time is piecewise exponential

$$f_{\text{i},aa}(t) = \begin{cases} \frac{2}{\theta_A} \mathrm{e}^{-\frac{2}{\theta_A}t}, & \text{if } 0 < t \le \tau_X, \\ \mathrm{e}^{-\frac{2}{\theta_A}\tau_X} \frac{2}{\theta_X} \mathrm{e}^{-\frac{2}{\theta_X}(t-\tau_X)}, & \text{if } \tau_X < t \le \tau_R, \\ \mathrm{e}^{-\frac{2}{\theta_A}\tau_X} \mathrm{e}^{-\frac{2}{\theta_X}(\tau_R-\tau_X)} \frac{2}{\theta_R} \mathrm{e}^{-\frac{2}{\theta_R}(t-\tau_R)}, & \text{if } t > \tau_R. \end{cases} \quad \text{(S2)}$$

This is independent of θ_B, θ_Y and φ.

Lastly, for two sequences from B, we have

$$f_{\text{i},bb}(t) = \begin{cases} \frac{2}{\theta_B} \mathrm{e}^{-\frac{2}{\theta_B}t}, & \text{if } 0 < t \le \tau_X, \\ \mathrm{e}^{-\frac{2}{\theta_B}\tau_X} \left[(1-\varphi)^2 \frac{2}{\theta_Y} \mathrm{e}^{-\frac{2}{\theta_Y}(t-\tau_X)} + \varphi^2 \frac{2}{\theta_X} \mathrm{e}^{-\frac{2}{\theta_X}(t-\tau_X)}\right], & \text{if } \tau_X < t \le \tau_R, \\ \mathrm{e}^{-\frac{2}{\theta_B}\tau_X} \left[(1-\varphi)^2 \mathrm{e}^{-\frac{2}{\theta_Y}(\tau_R-\tau_X)} + \varphi^2 \mathrm{e}^{-\frac{2}{\theta_X}(\tau_R-\tau_X)} + 2\varphi(1-\varphi)\right] \frac{2}{\theta_R} \mathrm{e}^{-\frac{2}{\theta_R}(t-\tau_R)}, & \text{if } t > \tau_R. \end{cases} \quad \text{(S3)}$$

This is independent of θ_A.

In this work, we always assume $\theta_A = \theta_X$ and $\theta_B = \theta_Y$, i.e., there is no change in the population size after introgression.

Density of coalescent time between two sequences under the MSC-M models (IM, IIM and SC) of figure 1b-d

In the IM model (fig. 1**b**), the two species (A and B) diverge at time τ_R and there is continuous gene flow from A to B at rate of $M_{A\to B}$ migrants per generation since τ_R. In the IIM model (fig. 1**c**) gene flow continues to occur from A to B after species divergence but stops at time $\tau_T > 0$ (Costa and Wilkinson-Herbots, 2017). In the SC model, (fig. 1**d**), the two species are initially completely isolated after their divergence but gene flow starts to occur from A to B at rate $M_{A\to B}$ from time $\tau_T > 0$ to the present (Costa and Wilkinson-Herbots, 2021). The IM model (fig. 1**b**) can be viewed as a special case of the IIM model (fig. 1**c**) with $\tau_T = 0$ or as a special case of the SC model (fig. 1**d**) with $\tau_T = \tau_R$. Here we derive the densities for the coalescent time between two sequences (t_{aa}, t_{ab}, t_{bb}) under the IIM and SC models.

Consider the IIM model of figure 1**b**. The parameter vector is $\boldsymbol{\theta}_{\text{IIM}} = \{M, \tau_T, \tau_R, \theta_A, \theta_B, \theta_T, \theta_R\}$. The backwards-in-time process of coalescent and migration in time interval (τ_T, τ_R) for two sequences is described by a Markov chain with four states: AB, AA, BB and A (Notohara, 1990). Here AA means two sequences in A and A means one sequence in A (i.e., a coalescent event has occurred, reducing the number of sequences from two to one). Note that the AB-to-AA transition means migration of a sequence from A to B in the real world (forward time). The rate matrix of this Markov chain is

$$Q = \begin{array}{c|cccc} & AA & AB & BB & A \\ \hline AA & -\frac{2}{\theta_A} & 0 & 0 & \frac{2}{\theta_A} \\ AB & w & -w & 0 & 0 \\ BB & 0 & 2w & -2w-\frac{2}{\theta_B} & \frac{2}{\theta_B} \\ A & 0 & 0 & 0 & 0 \end{array} \tag{S4}$$

where $w = m_{A\to B}/\mu = 4M_{A\to B}/\theta_B$ is the mutation-scaled migration rate; see, e.g., Herbots (1997), Wilkinson-Herbots (2008), Hobolth *et al.* (2011), and Jiao *et al.* (2020). Coalescence occurs at rate $\frac{2}{\theta_A}$ in A and $\frac{2}{\theta_B}$ in B. Time is measured in the expected number of mutations per site. The matrix Q has eigenvalues $\lambda_1 = 0$, $\lambda_2 = -\frac{2}{\theta_A}$, $\lambda_3 = -w$, and $\lambda_4 = -\frac{2}{\theta_B} - 2w$. Let the transition probability matrix over time t be $P(t) = \{p_{ij}(t)\} = \mathrm{e}^{Qt}$, where $p_{ij}(t)$ is the probability that the Markov chain will be in state j time t later given that it is in state i at time 0. This is

$$P(t) = \begin{bmatrix} \mathrm{e}^{-\frac{2}{\theta_A}t} & 0 & 0 & 1-\mathrm{e}^{-\frac{2}{\theta_A}t} \\ \frac{\theta_A w}{2-\theta_A w}\left(\mathrm{e}^{-wt}-\mathrm{e}^{-\frac{2}{\theta_A}t}\right) & \mathrm{e}^{-wt} & 0 & 1-\frac{2\,\mathrm{e}^{-wt}-\theta_A w\,\mathrm{e}^{-\frac{2}{\theta_A}t}}{2-\theta_A w} \\ h(t) & \frac{2\theta_B w}{2+\theta_B w}\left(\mathrm{e}^{-wt}-\mathrm{e}^{-\frac{2}{\theta_B+2w}t}\right) & \mathrm{e}^{-\frac{2}{\theta_B+2w}t} & g(t) \\ 0 & 0 & 0 & 1 \end{bmatrix}, \tag{S5}$$

where

$$h(t) = \frac{w^2\theta_A\theta_B}{(2-w\theta_A)(2+w\theta_B)(\theta_A-\theta_B+w\theta_A\theta_B)} \left[(2-w\theta_A)\theta_B e^{-(\frac{2}{\theta_B}+2w)t} - \theta_A(2+w\theta_B)e^{-\frac{2}{\theta_A}t} + 2(\theta_A-\theta_B+w\theta_A\theta_B)e^{-wt}\right] \tag{S6}$$

and

$$g(t) = 1 - h(t) - \frac{2\theta_B w}{2+\theta_B w}\left(\mathrm{e}^{-wt}-\mathrm{e}^{-\frac{2}{\theta_B+2w}t}\right) - \mathrm{e}^{-\frac{2}{\theta_B+2w}t}. \tag{S7}$$

Let $\tilde{P}(t)$ denote the transition probability matrix during (τ_T, τ_R). This is the same as $P(t)$ except that population T is the recipient of gene flow instead of B, with θ_B replaced by θ_T. Under the IIM model (fig. 1**c**), the probability density of the coalescent time t_{ab} is

$$\begin{aligned} f_{\text{IIM},ab}(t) &= \begin{cases} \tilde{P}_{AT,AA}(t-\tau_T)\frac{2}{\theta_A}, & \text{if } \tau_T < t \le \tau_R, \\ [1-\tilde{P}_{AT,A}(\tau_R-\tau_T)]\frac{2}{\theta_R}\,\mathrm{e}^{-\frac{2}{\theta_R}(t-\tau_R)}, & \text{if } t > \tau_R \end{cases} \\ &= \begin{cases} \frac{2w}{2-\theta_A w}\left[\mathrm{e}^{-w(t-\tau_T)} - \mathrm{e}^{-\frac{2}{\theta_A}(t-\tau_T)}\right], & \text{if } \tau_T < t \le \tau_R, \\ \left[\frac{2}{2-\theta_A w}\,\mathrm{e}^{-w(\tau_R-\tau_T)} - \frac{\theta_A w}{2-\theta_A w}\,\mathrm{e}^{-\frac{2}{\theta_A}(\tau_R-\tau_T)}\right]\frac{2}{\theta_R}\,\mathrm{e}^{-\frac{2}{\theta_R}(t-\tau_R)}, & \text{if } t > \tau_R. \end{cases} \end{aligned} \tag{S8}$$

This is independent of θ_B. Note that it is a function of $w = 4M_{AT}/\theta_T$ but not of M_{AT} and θ_T individually.

The other two coalescent times, t_{aa} and t_{bb}, have densities

$$\begin{aligned} f_{\text{IIM},aa}(t) &= \begin{cases} \frac{2}{\theta_A}\,\mathrm{e}^{-\frac{2}{\theta_A}t}, & \text{if } 0 < t \le \tau_T, \\ P_{AA,AA}(\tau_T)\left[\tilde{P}_{AA,AA}(t-\tau_T)\frac{2}{\theta_A} + \tilde{P}_{AA,TT}(t-\tau_T)\frac{2}{\theta_T}\right], & \text{if } \tau_T < t \le \tau_R, \\ P_{AA,AA}(\tau_T)\left[\tilde{P}_{AA,AA}(\tau_R-\tau_T) + \tilde{P}_{AA,TT}(\tau_R-\tau_T)\right]\frac{2}{\theta_R}\,\mathrm{e}^{-\frac{2}{\theta_R}(t-\tau_R)}, & \text{if } t > \tau_R \end{cases} \\ &= \begin{cases} \frac{2}{\theta_A}\,\mathrm{e}^{-\frac{2}{\theta_A}t}, & \text{if } 0 < t \le \tau_R, \\ \mathrm{e}^{-\frac{2}{\theta_A}(\tau_R-\tau_T)}\,\frac{2}{\theta_R}\,\mathrm{e}^{-\frac{2}{\theta_R}(t-\tau_R)}, & \text{if } t > \tau_R, \end{cases} \end{aligned} \tag{S9}$$

which depends on θ_A, θ_R, and τ_R only, and

$$
\begin{aligned}
f_{\text{IIM},bb}(t) &= \begin{cases}
\frac{2}{\theta_B}\,\mathrm{e}^{-\frac{2}{\theta_B}t}, & \text{if } 0 < t \le \tau_T,\\
\mathrm{e}^{-\frac{2}{\theta_B}\tau_T}\left[\tilde{P}_{TT,\,AA}(t-\tau_T)\frac{2}{\theta_A} + \tilde{P}_{TT,\,TT}(t-\tau_T)\frac{2}{\theta_T}\right], & \text{if } \tau_T < t \le \tau_R,\\
\mathrm{e}^{-\frac{2}{\theta_B}\tau_T}\left[1-\tilde{P}_{TT,\,A}(\tau_R-\tau_T)\right]\frac{2}{\theta_R}\,\mathrm{e}^{-\frac{2}{\theta_R}(t-\tau_R)}, & \text{if } t > \tau_R
\end{cases}\\
&= \begin{cases}
\frac{2}{\theta_B}\,\mathrm{e}^{-\frac{2}{\theta_B}t}, & \text{if } 0 < t \le \tau_T,\\
\mathrm{e}^{-\frac{2}{\theta_B}\tau_T}\left[\tilde{h}(t-\tau_T)\frac{2}{\theta_A} + \mathrm{e}^{-(\frac{2}{\theta_T}+2w)(t-\tau_T)}\ \frac{2}{\theta_T}\right], & \text{if } \tau_T < t \le \tau_R,\\
\mathrm{e}^{-\frac{2}{\theta_B}\tau_T}\left[1-\tilde{g}(\tau_R-\tau_T)\right]\frac{2}{\theta_R}\,\mathrm{e}^{-\frac{2}{\theta_R}(t-\tau_R)}, & \text{if } t > \tau_R,
\end{cases}
\end{aligned}
\tag{S10}
$$

which depends on all seven parameters in $\boldsymbol{\theta}_{\text{IIM}}$.

As $M \to \infty$, the IIM model becomes equivalent to the MSC-I model (fig. 1**a**) with $\varphi = 1$ provided $\tau_T = \tau_X$ and $\theta_T = \theta_X$. Also the IIM model with $M = 0$ and any value of τ_T is equivalent to the MSC-I model with $\varphi = 0$.

For the IM model (fig. 1**b**), the densities $f_{\text{IM},ab}(t)$, $f_{\text{IM},aa}(t)$ and $f_{\text{IM},bb}(t)$ are given by setting $\tau_T = 0$ and $w = 4M_{AB}/\theta_B$ in $f_{\text{IIM},ab}(t)$, $f_{\text{IIM},aa}(t)$ and $f_{\text{IIM},bb}(t)$, respectively.

Finally, under the SC model (fig. 1**c**), the parameter vector is $\boldsymbol{\theta}_{\text{SC}} = \{M, \tau_T, \tau_R, \theta_A, \theta_B, \theta_T, \theta_R\}$. The coalescent-with-migration process over the time interval (0, τ_T) is described by the Markov chain of eq. S4. The probability density of the coalescent time t_{ab} is

$$
\begin{aligned}
f_{\text{SC},ab}(t) &= \begin{cases}
P_{AB,\,AA}(t)\frac{2}{\theta_A}, & \text{if } 0 < t < \tau_T,\\
P_{AB,\,AA}(\tau_T)\frac{2}{\theta_A}\,\mathrm{e}^{-\frac{2}{\theta_A}(t-\tau_T)}, & \text{if } \tau_T < t < \tau_R,\\
\left[P_{AB,\,AA}(\tau_T)\,\mathrm{e}^{-\frac{2}{\theta_A}(\tau_R-\tau_T)} + P_{AB,\,AB}(\tau_T)\right]\times\frac{2}{\theta_R}\,\mathrm{e}^{-\frac{2}{\theta_R}(t-\tau_R)}, & \text{if } t > \tau_R
\end{cases}\\
&= \begin{cases}
\frac{w\theta_A}{2-w\theta_A}\left[\mathrm{e}^{-wt}-\mathrm{e}^{-\frac{2}{\theta_A}t}\right]\frac{2}{\theta_A}, & \text{if } 0 < t < \tau_T,\\
\frac{w\theta_A}{2-w\theta_A}\left[\mathrm{e}^{-w\tau_T}-\mathrm{e}^{-\frac{2}{\theta_A}\tau_T}\right]\frac{2}{\theta_A}\,\mathrm{e}^{-\frac{2}{\theta_A}(t-\tau_T)}, & \text{if } \tau_T < t < \tau_R,\\
\left[\frac{w\theta_A}{2-w\theta_A}\left[\mathrm{e}^{-w\tau_T}-\mathrm{e}^{-\frac{2}{\theta_A}\tau_T}\right]\mathrm{e}^{-\frac{2}{\theta_A}(\tau_R-\tau_T)} + \mathrm{e}^{-w\tau_T}\right]\times\frac{2}{\theta_R}\,\mathrm{e}^{-\frac{2}{\theta_R}(t-\tau_R)}, & \text{if } t > \tau_R,
\end{cases}
\end{aligned}
\tag{S11}
$$

which is independent of θ_B and θ_T. The coalescent time t_{aa} has density

$$
\begin{aligned}
f_{\text{SC},aa}(t) &= \begin{cases}
P_{AA,\,AA}(t)\frac{2}{\theta_A}, & \text{if } 0 < t < \tau_T,\\
P_{AA,\,AA}(\tau_T)\frac{2}{\theta_A}\,\mathrm{e}^{-\frac{2}{\theta_A}(t-\tau_T)}, & \text{if } \tau_T < t < \tau_R,\\
\left[P_{AA,\,AA}(\tau_T)\,\mathrm{e}^{-\frac{2}{\theta_A}(\tau_R-\tau_T)}\right]\times\frac{2}{\theta_R}\,\mathrm{e}^{-\frac{2}{\theta_R}(t-\tau_R)}, & \text{if } t > \tau_R
\end{cases}\\
&= \begin{cases}
\frac{2}{\theta_A}\,\mathrm{e}^{-\frac{2}{\theta_A}t}, & \text{if } 0 < t \le \tau_R,\\
\mathrm{e}^{-\frac{2}{\theta_A}(\tau_R-\tau_T)}\ \frac{2}{\theta_R}\,\mathrm{e}^{-\frac{2}{\theta_R}(t-\tau_R)}, & \text{if } t > \tau_R,
\end{cases}
\end{aligned}
\tag{S12}
$$

which is independent of θ_B and θ_T. The density is identical to $f_{\text{IIM},aa}(t)$.

Lastly, the coalescent time t_{bb} has density

$$
\begin{aligned}
f_{\text{SC},ab}(t) &= \begin{cases}
P_{BB,\,AA}(t)\frac{2}{\theta_A} + P_{BB,\,BB}(t)\frac{2}{\theta_B}, & \text{if } 0 < t < \tau_T,\\
P_{BB,\,AA}(\tau_T)\frac{2}{\theta_A}\,\mathrm{e}^{-\frac{2}{\theta_A}(t-\tau_T)} + P_{BB,\,BB}(\tau_T)\frac{2}{\theta_T}\,\mathrm{e}^{-\frac{2}{\theta_T}(t-\tau_T)}, & \text{if } \tau_T < t < \tau_R,\\
\left[P_{BB,\,AA}(\tau_T)\,\mathrm{e}^{-\frac{2}{\theta_A}(\tau_R-\tau_T)} + P_{BB,\,AB}(\tau_T) + P_{BB,\,BB}(\tau_T)\,\mathrm{e}^{-\frac{2}{\theta_T}(\tau_R-\tau_T)}\right]\times\frac{2}{\theta_R}\,\mathrm{e}^{-\frac{2}{\theta_R}(t-\tau_R)}, & \text{if } t > \tau_R
\end{cases}\\
&= \begin{cases}
h(t)\frac{2}{\theta_A} + \mathrm{e}^{-(\frac{2}{\theta_B}+2w)t}\ \frac{2}{\theta_B}, & \text{if } 0 < t < \tau_T,\\
h(\tau_T)\frac{2}{\theta_A}\,\mathrm{e}^{-\frac{2}{\theta_A}(t-\tau_T)} + \mathrm{e}^{-(\frac{2}{\theta_B}+2w)\tau_T}\ \frac{2}{\theta_T}\,\mathrm{e}^{-\frac{2}{\theta_T}(t-\tau_T)}, & \text{if } \tau_T < t < \tau_R,\\
\Big[h(\tau_T)\,\mathrm{e}^{-\frac{2}{\theta_A}(\tau_R-\tau_T)} + \frac{2w\theta_B}{2+w\theta_B}\left\{\mathrm{e}^{-w\tau_T}-\mathrm{e}^{-(\frac{2}{\theta_B}+2w)\tau_T}\right\} + \mathrm{e}^{-(\frac{2}{\theta_B}+2w)\tau_T}\,\mathrm{e}^{-\frac{2}{\theta_T}(\tau_R-\tau_T)}\Big] & \\
\quad\times\frac{2}{\theta_R}\,\mathrm{e}^{-\frac{2}{\theta_R}(t-\tau_R)}, & \text{if } t > \tau_R,
\end{cases}
\end{aligned}
\tag{S13}
$$

which depends on all seven parameters in $\boldsymbol{\theta}_{\text{SC}}$.

References

Costa, R. J. and Wilkinson-Herbots, H. 2017. Inference of gene flow in the process of speciation: An efficient maximum-likelihood method for the isolation-with-initial-migration model. *Genetics*, 205(4): 1597–1618.

Costa, R. J. and Wilkinson-Herbots, H. M. 2021. Inference of gene flow in the process of speciation: Efficient maximum-likelihood implementation of a generalised isolation-with-migration model. *Theor. Popul. Biol.*, 140(1–15).

Herbots, H. M. 1997. The structured coalescent. In *Progress in population genetics and human evolution*, volume 87 of *IMA Vol. Math. Appl.*, pages 231–255.

Hobolth, A., Andersen, L. N., and Mailund, T. 2011. On computing the coalescence time density in an isolation-with-migration model with few samples. *Genetics*, 187(4): 1241–1243.

Ji, J., Jackson, D. J., Leaché, A. D., and Yang, Z. 2023. Power of Bayesian and heuristic tests to detect cross-species introgression with reference to gene flow in the *Tamias quadrivittatus* group of North American chipmunks. *Syst. Biol.*, 72(2): 446–465.

Jiao, X., Flouri, T., Rannala, B., and Yang, Z. 2020. The impact of cross-species gene flow on species tree estimation. *Syst. Biol.*, 69(5): 830–847.

Notohara, M. 1990. The coalescent and the genealogical process in geographically structured populations. *J. Math. Biol.*, 29: 59–75.

Wilkinson-Herbots, H. M. 2008. The distribution of the coalescence time and the number of pairwise nucleotide differences in the "isolation with migration" model. *Theor. Popul. Biol.*, 73: 277–288.

Yang, Z. and Flouri, T. 2022. Estimation of cross-species introgression rates using genomic data despite model unidentifiability. *Mol. Biol. Evol.*, 39(5): msac083.

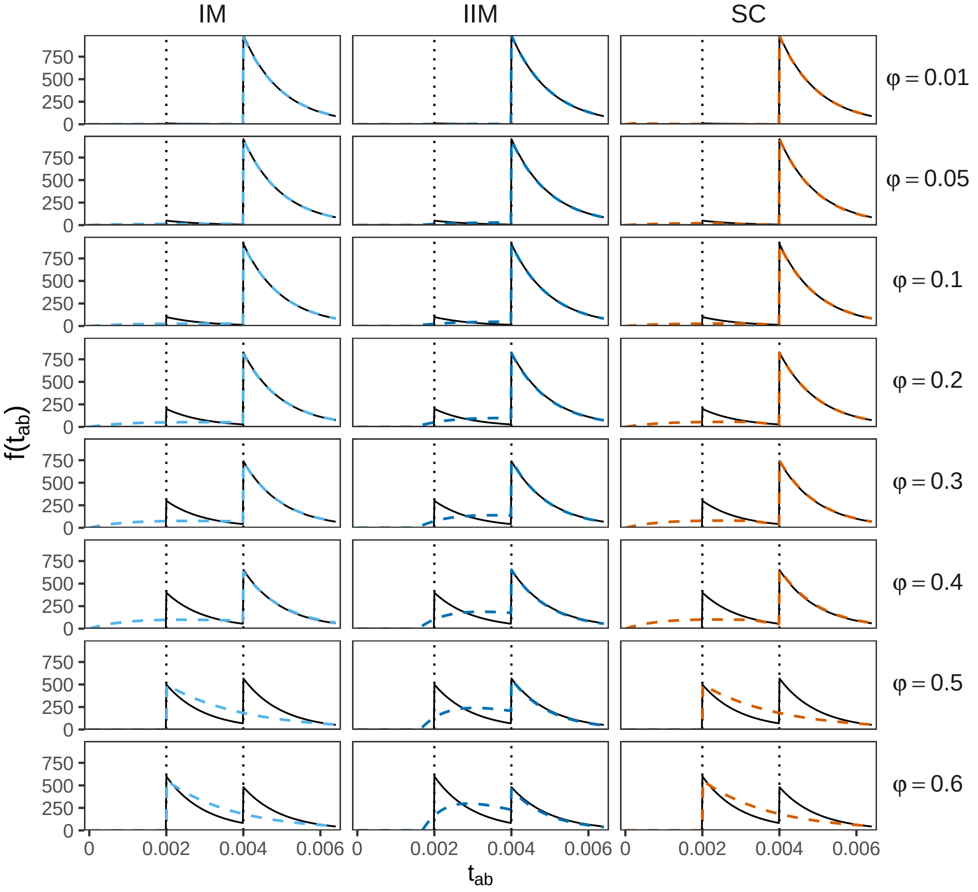

Fig. S1: [kl-$f_{ab}(t)$] Distributions of the coalescent time t_{ab} between a sequence from A and a sequence from B under models of figure 1. The true distribution under the MSC-I model of figure 1**a** for different values of introgression probability φ is shown in solid curve, while the best-fitting distributions under the MSC-M models (IM, IIM, SC) of figure 1**b–d**), calculated by minimizing the KL divergence from the true MSC-I model (eq. 3) under the constraint $\theta_A = \theta_R$, are shown as dashed curves (see fig. 2).

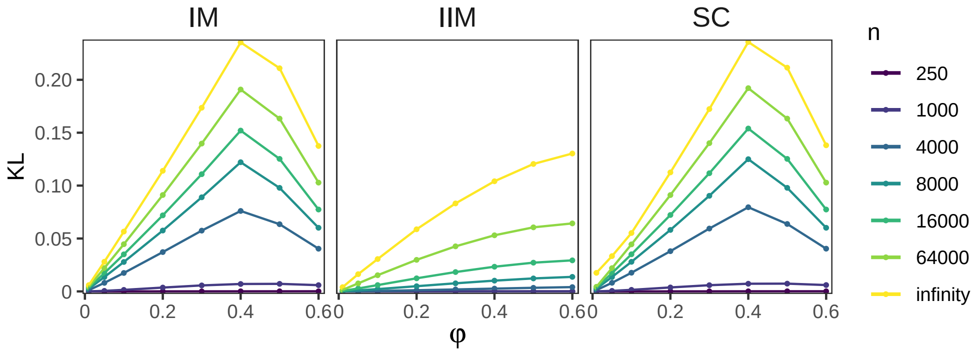

Fig. S2: [kl-vs-φ] KL divergence (eq. 3) for the IM, IIM and SC in figure 1**b–d**. Lower values indicate a better fit. Parameter estimates are in figure 2.

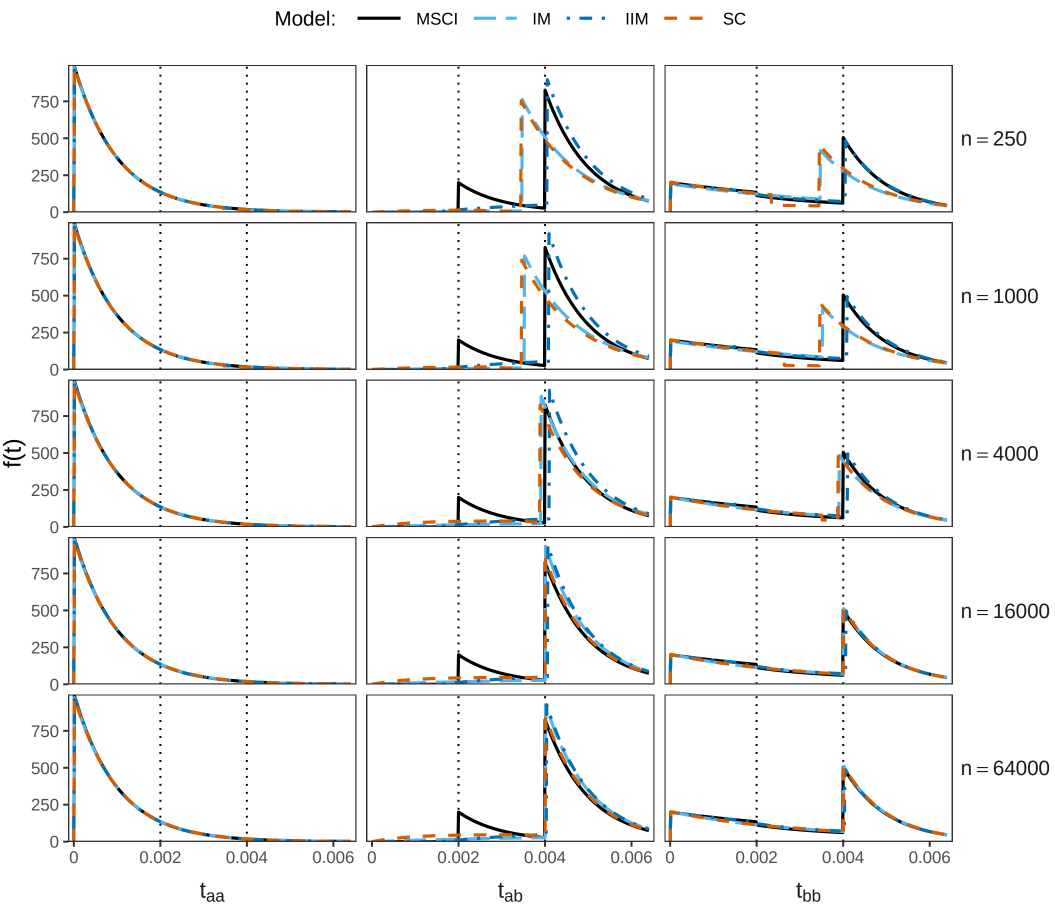

Fig. S3: [2s-ft-*n*] Distributions of coalescent times between two sequences (t_{aa}, t_{ab} and t_{bb}) under the true MSC-I model (fig. 1**a**) with different numbers of sites per locus (n) and under the MSC-M models (IM, IIM, SC; fig. 1**b**-**d**) calculated using the best-fitting parameter values obtained from BPP analysis of data of L = 4,000 loci (fig. 3, first column). See legend to figure S6.

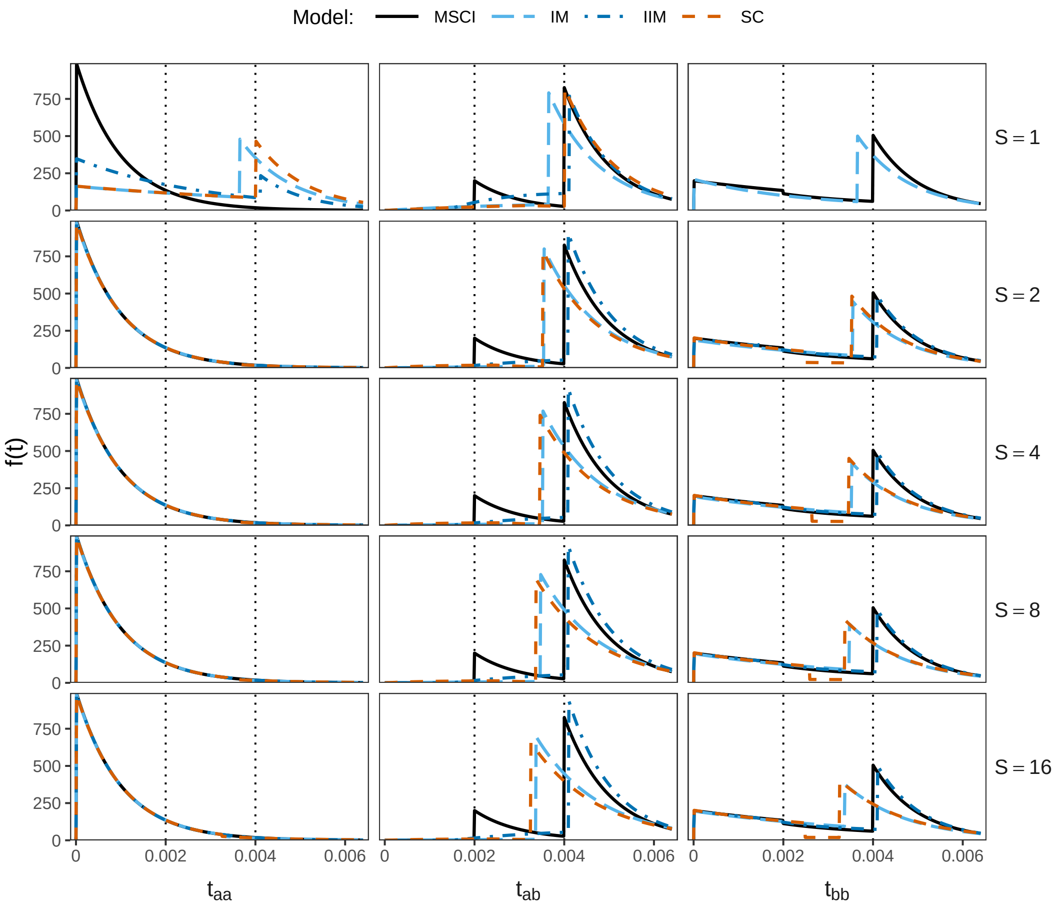

Fig. S4: [2s-ft-*S*] Distributions of coalescent times between two sequences (t_{aa}, t_{ab} and t_{bb}) under the true MSC-I model (fig. 1**a**) at different numbers of sequences per species (S) and under the three fitting MSC-M models (IM, IIM, SC; fig. 1**b**-**d**) calculated using the best-fitting parameter values obtained from BPP analysis of data of L = 4,000 loci (fig. 3, second column).

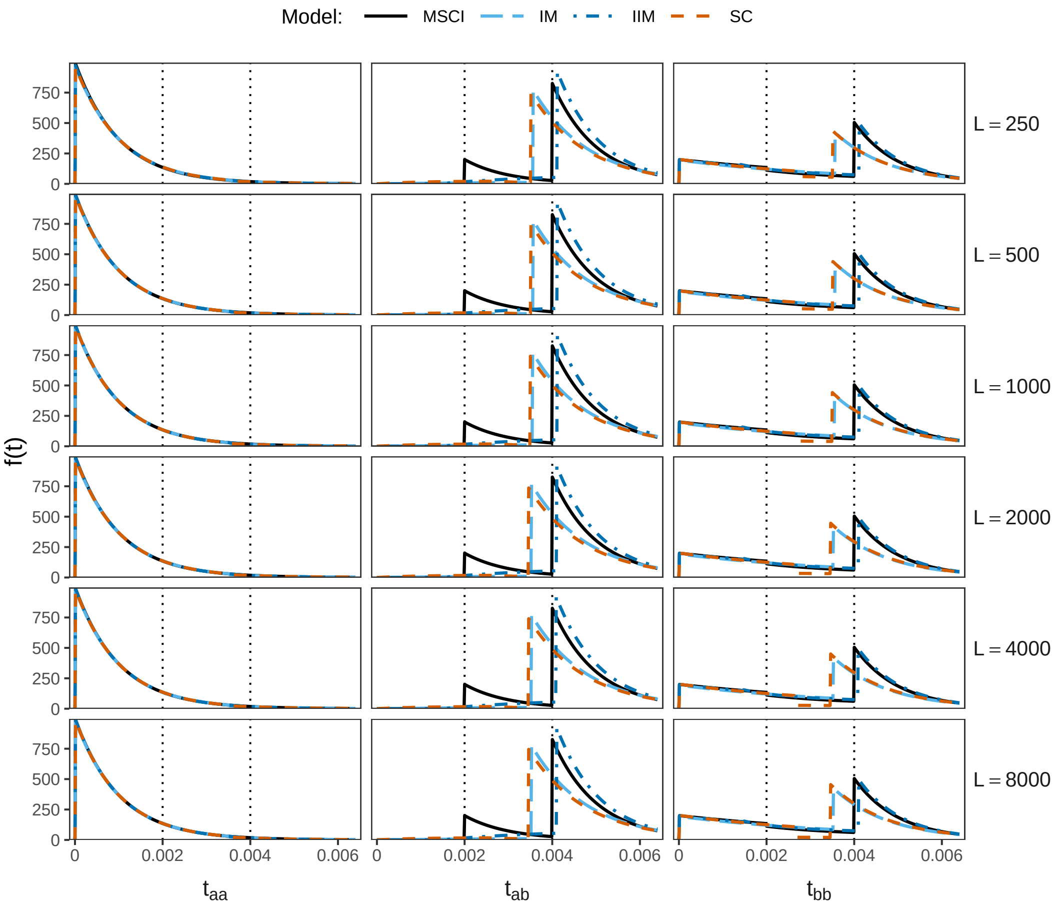

Fig. S5: [2s-ft-*L*] Distributions of coalescent times between two sequences (t_{aa}, t_{ab} and t_{bb}) under the true MSC-I model (fig. 1**a**) at different numbers of loci (L) and under the three fitting MSC-M models (IM, IIM, SC; fig. 1**b**-**d**) using the best-fitting parameter values obtained from BPP (fig. 3, third column). Note that different choices of L led to highly similar estimates and coalescent time distributions.

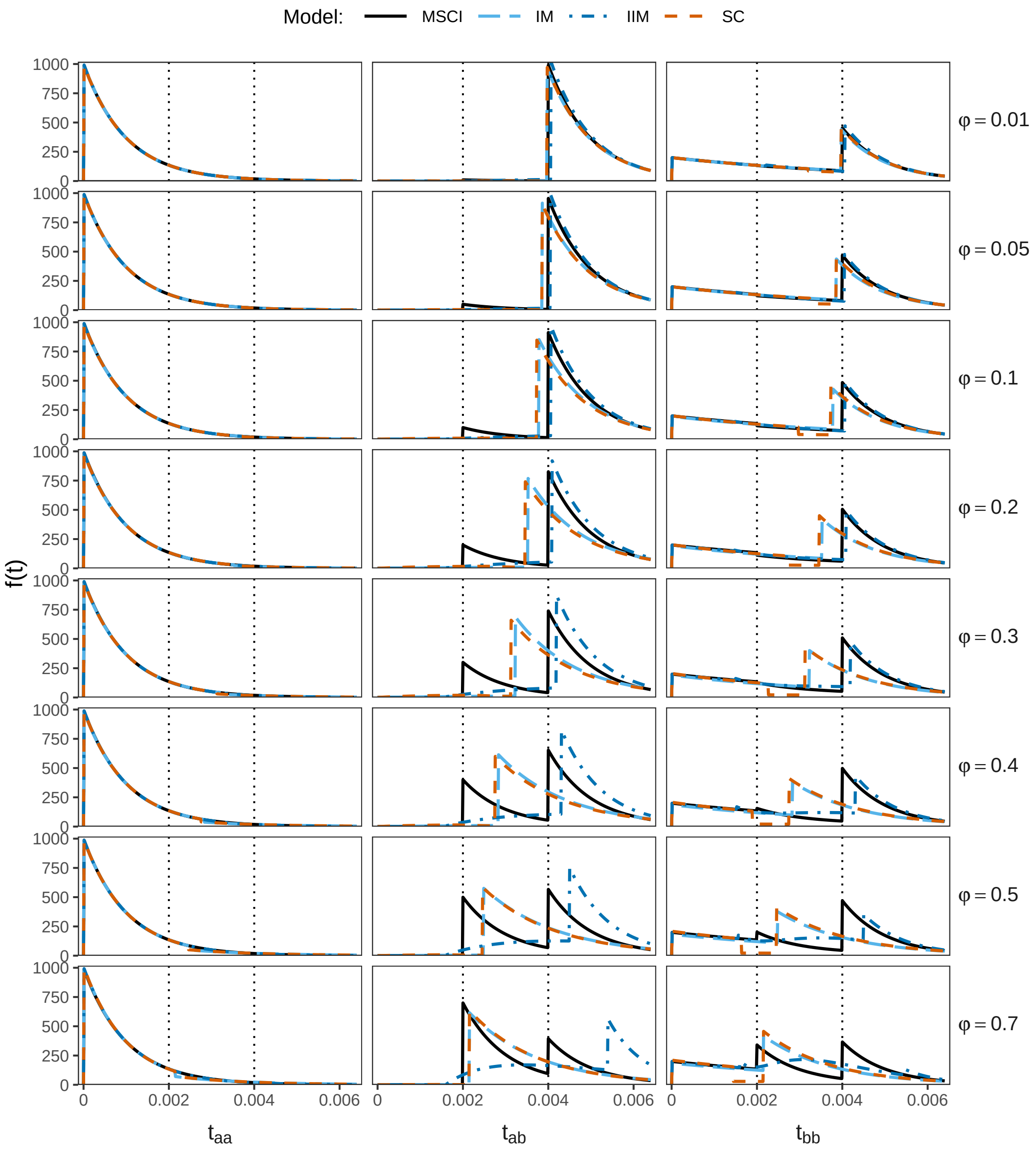

Fig. S6: [2s-ft-φ] Distributions of coalescent times between two sequences (t_{aa}, t_{ab} and t_{bb}) under the true MSC-I model (fig. 1**a**) at different values of the introgression probability (φ; solid curves) and under the three fitting MSC-M models (IM, IIM, SC in fig. 1**b-d**; dashed curves) calculated using the approximate best-fitting parameter values obtained from BPP for data of L = 4,000 loci, each with S = 4 sequences per species and with n = 1,000 sites per sequence (fig. 3, last column). The two vertical dotted lines indicate the introgression time (τ_X = 0.002) and the divergence time (τ_R = 0.004).

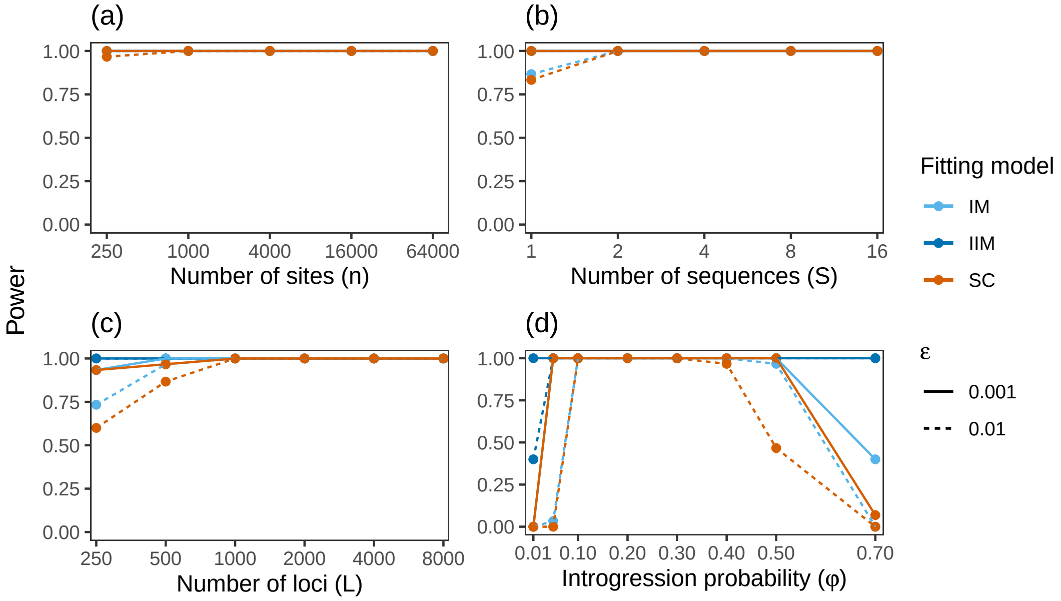

Fig. S7: [2s-bf] Power of the Bayesian test for gene flow applied to data simulated under the MSC-I model of figure 1**a**. The Bayes factor B_{10} for comparing the null model $H_0 : M = 0$ and the alternative model $H_1 : M > 0$ under the IM, IIM or SC models (fig. 1**b**-**d**) is calculated using the Savage-Dickey density ratio, with the null region defined as $M < \epsilon = 0.001$ or 0.01 (Ji *et al.*, 2023). Power is the proportion of replicate datasets in which $B_{10} > 100$ (as the cutoff is 100, we used $\epsilon = 0.001$). Parameter estimates for those data are summarized in figure 3.

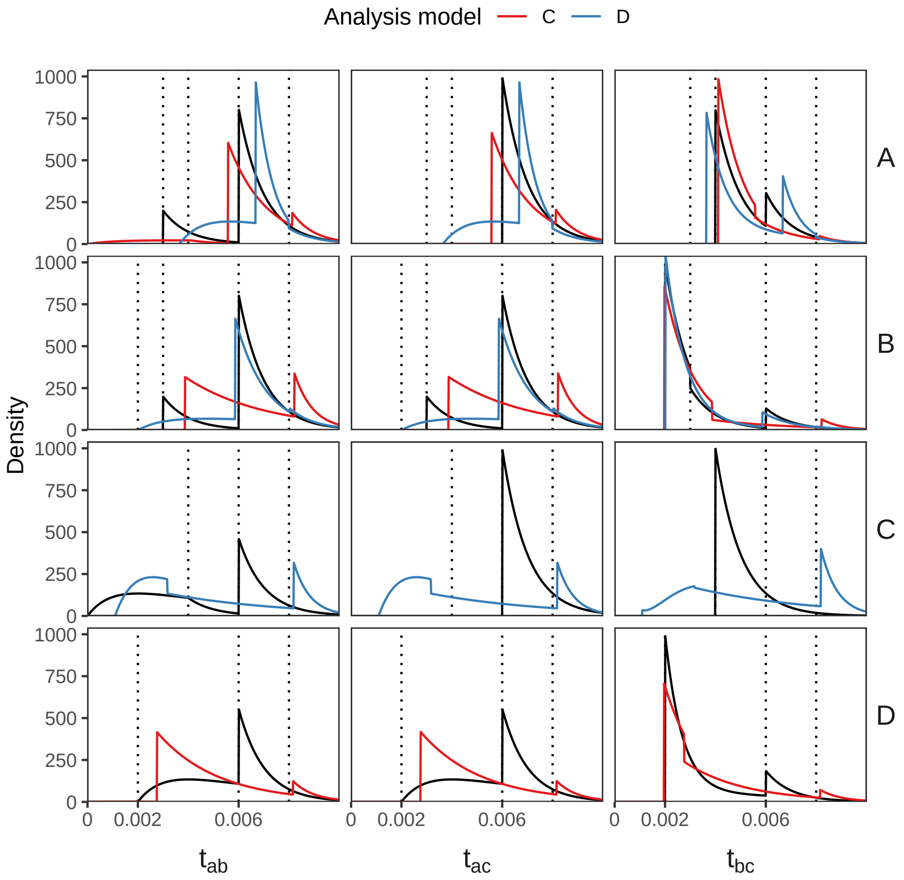

Fig. S8: [4s-ft] The true (black) and fitting (red and blue) distributions of coalescent times between sequences from two species, $f(t_{ab})$, $f(t_{ac})$, $f(t_{bc})$, when data are generated under models A, B, C, and D of figure 4 and analyzed under models C and D. Each row corresponds to a true model (A, B, C, or D). Each colour line indicates the fitting model (C or D). For example, the first row shows the A-C (red) and A-D (blue) settings (fig. 4). The fitting distributions were calculated using posterior means of parameters from BPP analysis of simulated data of $L = 4{,}000$ loci, averaged across 100 replicates (fig. 4**e**).

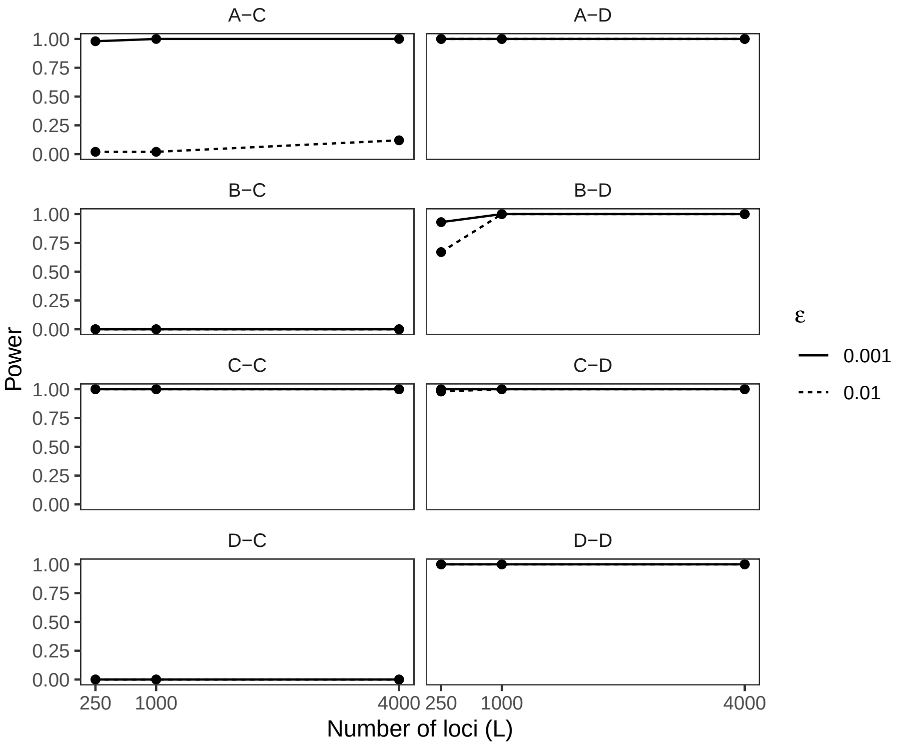

Fig. S9: [4s-ABCD-bf] Power of the Bayesian test of gene flow applied to data simulated under models A-D of figure 4**a–d** (see fig. 4**e** for parameter estimates). Bayes factor is calculated to test the null hypothesis $H_0 : M = 0$ against $H_1 : M > 0$. See legend to figure S7 for more details.

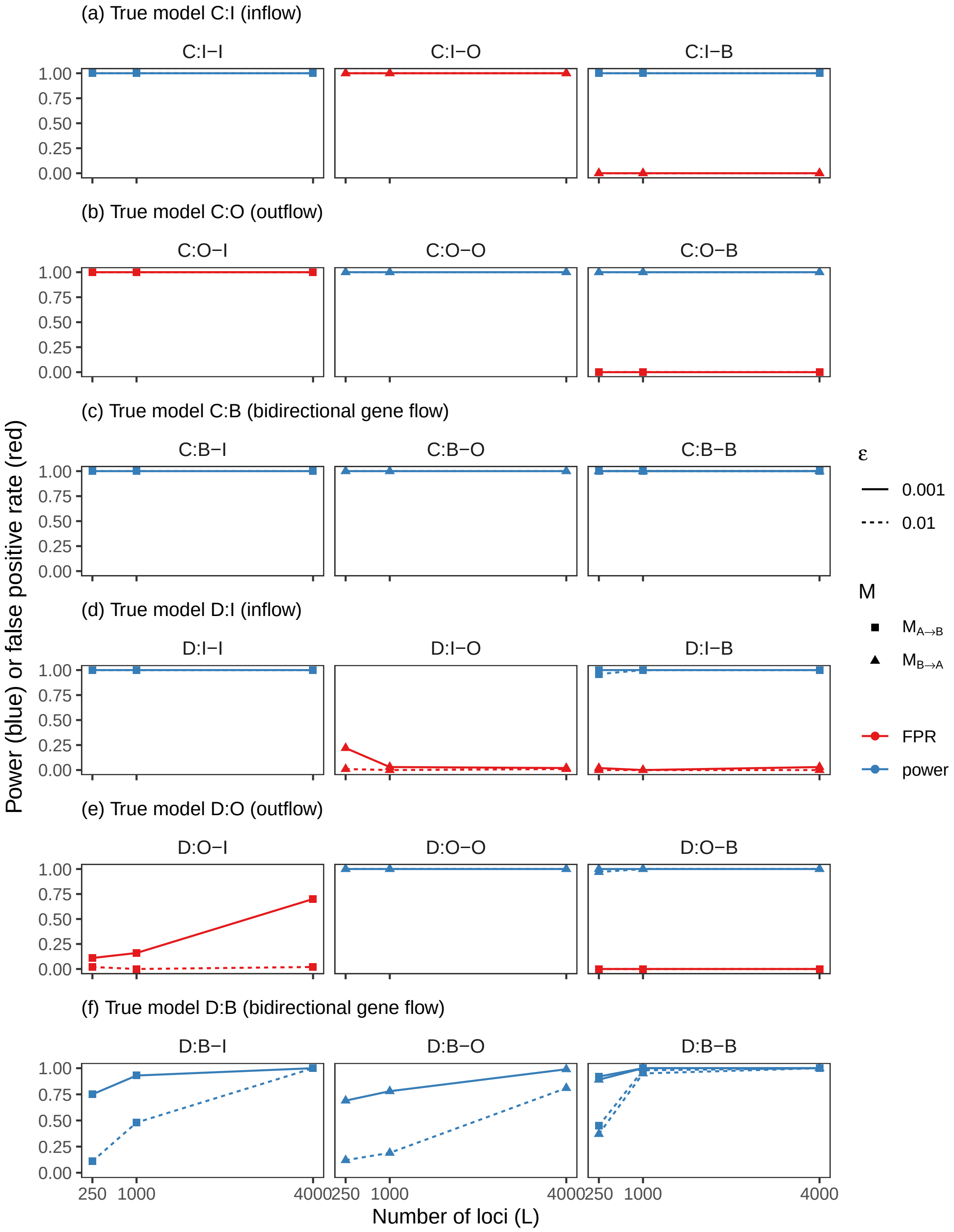

Fig. S10: [4s-IOB-bf] Power (blue) and false positive rate (FPR; red) of Bayesian test of gene flow applied to data of four species simulated under the C and D models: (a) C:I (inflow), (b) C:O (outflow), (c) C:B (bidirectional), (d) D:I (inflow), (e) D:O (outflow), (f) D:B (bidirectional) (fig. 5**a**-**f**). The data were analyzed under I, O, and B models. Parameter estimates are shown in figure 5**g**-**h**. See legend to figure S7 for more details.

Table S1: Posterior means and 95% HPD intervals (in parentheses) for parameters, averaged over 100 replicated datasets, obtained from BPP analysis of simulated datasets of L = 4,000 loci for the eight simulation-analysis model settings in figure 4**e**

	A-C	B-C	C-C	D-C	A-D	B-D	C-D	D-D
θ_A	1.99 (1.93, 2.04)	1.96 (1.90, 2.02)	2.00 (1.94, 2.05)	1.91 (1.86, 1.97)	2.00 (1.94, 2.06)	2.00 (1.94, 2.06)	2.18 (2.12, 2.24)	2.00 (1.94, 2.05)
θ_B	1.92 (1.87, 1.98)	1.99 (1.93, 2.05)	2.01 (1.94, 2.08)	1.98 (1.92, 2.05)	2.06 (2.00, 2.12)	2.01 (1.94, 2.07)	2.61 (2.52, 2.69)	2.00 (1.94, 2.06)
θ_C	2.00 (1.94, 2.06)	2.00 (1.94, 2.06)	2.00 (1.94, 2.06)	2.00 (1.94, 2.06)	1.93 (1.88, 1.99)	2.01 (1.94, 2.07)	1.12 (1.09, 1.16)	2.00 (1.94, 2.06)
θ_D	2.00 (1.94, 2.06)	1.99 (1.93, 2.05)	2.00 (1.94, 2.06)	1.99 (1.93, 2.05)	2.00 (1.94, 2.06)	2.00 (1.94, 2.06)	1.97 (1.91, 2.03)	2.00 (1.94, 2.05)
θ_R	1.75 (1.48, 2.02)	1.48 (1.17, 1.79)	1.99 (1.71, 2.26)	1.65 (1.32, 1.97)	2.04 (1.76, 2.32)	1.96 (1.65, 2.26)	1.30 (0.99, 1.61)	1.99 (1.66, 2.32)
θ_S	2.99 (2.64, 3.34)	6.32 (5.91, 6.72)	2.03 (1.68, 2.38)	4.77 (4.57, 4.98)	1.36 (0.94, 1.79)	2.36 (1.76, 2.98)	9.29 (8.80, 9.78)	2.03 (1.25, 2.77)
θ_T	2.02 (1.72, 2.32)	2.30 (2.15, 2.45)	2.05 (1.58, 2.52)	2.83 (2.50, 3.16)	2.55 (2.24, 2.86)	1.92 (1.78, 2.05)	58.46 (49.94, 67.25)	2.00 (1.85, 2.16)
τ_R	8.12 (7.97, 8.27)	8.21 (8.04, 8.38)	8.00 (7.85, 8.15)	8.15 (7.97, 8.33)	7.98 (7.83, 8.13)	8.02 (7.86, 8.19)	8.18 (8.01, 8.35)	8.01 (7.83, 8.18)
τ_S	5.57 (5.44, 5.70)	3.87 (3.78, 3.97)	6.00 (5.85, 6.15)	2.75 (2.69, 2.81)	6.67 (6.42, 6.92)	5.86 (5.56, 6.17)	3.17 (3.04, 3.29)	5.98 (5.31, 6.66)
τ_T	4.11 (4.00, 4.22)	1.97 (1.91, 2.02)	3.99 (3.85, 4.13)	1.96 (1.91, 2.02)	3.65 (3.55, 3.74)	2.02 (1.97, 2.07)	1.09 (1.06, 1.12)	2.00 (1.95, 2.05)
M	0.01 (0.01, 0.01)	0.00 (0.00, 0.00)	0.20 (0.19, 0.21)	0.00 (0.00, 0.00)	0.13 (0.12, 0.14)	0.04 (0.04, 0.04)	6.80 (5.75, 7.88)	0.20 (0.19, 0.21)

Note.— Values of τ and θ are multiplied by 10^3. Results for all data sizes (L = 250, 1000, 4000 loci) are shown in figure 4**e**.

Table S2: Posterior means and 95% HPD intervals (in parentheses) for parameters, averaged over 100 replicated datasets, obtained from BPP analysis of simulated datasets of $L = 4000$ loci for the nine simulation-analysis model settings for model C of figure 5**a**-**c**

	Model C:I				Model C:O				Model C:B			
	θ_I	$\hat{\theta}_I$ (C:I-I)	$\hat{\theta}_O$ (C:I-O)	$\hat{\theta}_B$ (C:I-B)	θ_O	$\hat{\theta}_I$ (C:O-I)	$\hat{\theta}_O$ (C:O-O)	$\hat{\theta}_B$ (C:O-B)	θ_B	$\hat{\theta}_I$ (C:B-I)	$\hat{\theta}_O$ (C:B-O)	$\hat{\theta}_B$ (C:B-B)
θ_A	2.0	2.00 (1.94, 2.05)	0.87 (0.83, 0.91)	1.99 (1.93, 2.04)	2.0	3.84 (3.74, 3.93)	2.00 (1.93, 2.07)	2.00 (1.93, 2.07)	2.0	3.60 (3.51, 3.68)	1.28 (1.22, 1.34)	2.00 (1.91, 2.08)
θ_B	2.0	2.01 (1.94, 2.08)	3.73 (3.64, 3.83)	2.01 (1.94, 2.08)	2.0	0.92 (0.88, 0.95)	2.00 (1.94, 2.06)	1.99 (1.93, 2.05)	2.0	1.29 (1.24, 1.35)	3.43 (3.34, 3.52)	2.00 (1.92, 2.08)
θ_C	2.0	2.00 (1.94, 2.06)	1.95 (1.90, 2.01)	2.00 (1.94, 2.06)	2.0	2.01 (1.95, 2.07)	2.00 (1.94, 2.06)	2.00 (1.95, 2.06)	2.0	2.00 (1.93, 2.06)	1.91 (1.85, 1.96)	2.00 (1.94, 2.06)
θ_D	2.0	2.00 (1.94, 2.06)	2.00 (1.94, 2.06)	2.00 (1.94, 2.06)	2.0	2.00 (1.94, 2.06)	2.00 (1.94, 2.06)	2.00 (1.94, 2.06)	2.0	1.99 (1.93, 2.05)	2.00 (1.94, 2.06)	2.00 (1.94, 2.06)
θ_R	2.0	1.99 (1.71, 2.26)	1.78 (1.48, 2.07)	2.00 (1.72, 2.28)	2.0	1.74 (1.42, 2.06)	2.00 (1.68, 2.31)	2.00 (1.68, 2.31)	2.0	1.51 (1.19, 1.82)	1.78 (1.47, 2.08)	1.98 (1.68, 2.28)
θ_S	2.0	2.03 (1.68, 2.38)	1.81 (0.95, 2.67)	1.99 (1.63, 2.34)	2.0	3.57 (3.37, 3.78)	2.01 (1.54, 2.49)	2.02 (1.55, 2.50)	2.0	5.26 (4.95, 5.57)	3.27 (2.73, 3.78)	2.08 (1.56, 2.60)
θ_T	2.0	2.05 (1.58, 2.52)	9.40 (8.12, 10.78)	2.05 (1.59, 2.53)	2.0	0.45 (0.14, 0.76)	2.02 (1.79, 2.25)	1.98 (1.74, 2.21)	2.0	0.33 (0.06, 0.63)	16.91 (10.24, 25.32)	2.04 (1.60, 2.50)
τ_R	8.0	8.00 (7.85, 8.15)	8.09 (7.94, 8.25)	8.00 (7.85, 8.15)	8.0	8.11 (7.94, 8.29)	8.01 (7.84, 8.17)	8.01 (7.84, 8.17)	8.0	8.18 (8.01, 8.35)	8.09 (7.93, 8.25)	8.01 (7.85, 8.17)
τ_S	6.0	6.00 (5.85, 6.15)	6.80 (6.30, 7.30)	6.02 (5.87, 6.18)	6.0	3.73 (3.65, 3.80)	6.00 (5.75, 6.25)	6.00 (5.75, 6.25)	6.0	3.76 (3.68, 3.85)	4.89 (4.48, 5.34)	5.95 (5.66, 6.26)
τ_T	4.0	3.99 (3.85, 4.13)	4.08 (3.97, 4.18)	3.99 (3.85, 4.13)	4.0	3.49 (3.34, 3.63)	3.99 (3.91, 4.08)	4.01 (3.92, 4.10)	4.0	3.58 (3.42, 3.73)	3.33 (3.22, 3.43)	3.99 (3.86, 4.11)
$M_{A\to B}$	0.2	0.20 (0.19, 0.21)	n/a	0.20 (0.19, 0.21)	n/a	0.17 (0.16, 0.17)	n/a	0.00 (0.00, 0.00)	0.2	0.34 (0.33, 0.36)	n/a	0.20 (0.19, 0.21)
$M_{B\to A}$	n/a	n/a	0.18 (0.17, 0.18)	0.01 (0.00, 0.02)	0.2	n/a	0.20 (0.19, 0.21)	0.20 (0.19, 0.21)	0.2	n/a	0.35 (0.33, 0.37)	0.20 (0.19, 0.21)

Note.— Values of τ and θ are multiplied by 10^3. Results for all data sizes (L = 250, 1000, 4000 loci) are shown in figure 5**g**. 'n/a' indicates that the parameter does not exist in the model.

Table S3: Posterior means and 95% HPD intervals (in parentheses) for parameters, averaged over 100 replicated datasets, obtained from BPP analysis of simulated datasets of L = 4000 loci for the nine simulation-analysis model settings for model D of figure 5**d-f**

	Model D:I				Model D:O				Model D:B			
	θ_I	$\hat\theta_I$ (D:I-I)	$\hat\theta_O$ (D:I-O)	$\hat\theta_B$ (D:I-B)	θ_O	$\hat\theta_I$ (D:O-I)	$\hat\theta_O$ (D:O-O)	$\hat\theta_B$ (D:O-B)	θ_B	$\hat\theta_I$ (D:B-I)	$\hat\theta_O$ (D:B-O)	$\hat\theta_B$ (D:B-B)
θ_A	2.0	2.00 (1.94, 2.05)	1.91 (1.85, 1.97)	1.99 (1.93, 2.04)	2.0	2.12 (2.06, 2.18)	2.00 (1.94, 2.06)	2.00 (1.95, 2.06)	2.0	2.05 (1.99, 2.11)	1.98 (1.92, 2.04)	2.00 (1.94, 2.06)
θ_B	2.0	2.00 (1.94, 2.06)	2.00 (1.94, 2.06)	2.00 (1.94, 2.06)	2.0	2.02 (1.96, 2.08)	2.00 (1.94, 2.06)	2.01 (1.94, 2.07)	2.0	1.99 (1.93, 2.06)	1.99 (1.93, 2.05)	1.99 (1.93, 2.06)
θ_C	2.0	2.00 (1.94, 2.06)	2.00 (1.93, 2.06)	2.00 (1.94, 2.07)	2.0	2.02 (1.96, 2.08)	2.00 (1.94, 2.06)	2.00 (1.94, 2.06)	2.0	2.00 (1.94, 2.06)	1.99 (1.93, 2.06)	2.00 (1.94, 2.06)
θ_D	2.0	2.00 (1.94, 2.05)	2.00 (1.94, 2.06)	2.00 (1.94, 2.06)	2.0	2.00 (1.94, 2.06)	2.00 (1.95, 2.06)	2.00 (1.95, 2.06)	2.0	2.00 (1.94, 2.06)	2.00 (1.94, 2.06)	2.00 (1.94, 2.06)
θ_R	2.0	1.99 (1.66, 2.32)	1.59 (1.24, 1.92)	2.00 (1.67, 2.33)	2.0	1.80 (1.47, 2.12)	1.99 (1.65, 2.32)	2.00 (1.67, 2.33)	2.0	1.68 (1.34, 2.02)	1.69 (1.35, 2.02)	1.98 (1.65, 2.31)
θ_S	2.0	2.03 (1.25, 2.77)	4.80 (4.59, 5.00)	2.00 (1.25, 2.73)	2.0	4.01 (3.80, 4.22)	1.92 (1.17, 2.63)	1.81 (1.06, 2.52)	2.0	4.59 (4.37, 4.81)	4.44 (4.13, 4.72)	2.16 (1.34, 2.96)
θ_T	2.0	2.00 (1.85, 2.16)	2.86 (2.52, 3.19)	2.01 (1.85, 2.17)	2.0	1.25 (1.11, 1.39)	2.00 (1.90, 2.10)	1.94 (1.82, 2.05)	2.0	1.86 (1.59, 2.14)	2.69 (2.26, 3.12)	2.04 (1.85, 2.25)
τ_R	8.0	8.01 (7.83, 8.18)	8.18 (8.00, 8.36)	8.00 (7.83, 8.17)	8.0	8.08 (7.90, 8.25)	8.00 (7.83, 8.18)	8.00 (7.82, 8.17)	8.0	8.14 (7.96, 8.32)	8.14 (7.96, 8.32)	8.01 (7.84, 8.19)
τ_S	6.0	5.98 (5.31, 6.66)	2.76 (2.69, 2.83)	5.98 (5.34, 6.63)	6.0	3.08 (2.99, 3.17)	6.07 (5.44, 6.72)	6.13 (5.51, 6.78)	6.0	2.82 (2.67, 2.98)	3.04 (2.78, 3.37)	5.85 (5.14, 6.56)
τ_T	2.0	2.00 (1.95, 2.05)	1.96 (1.90, 2.02)	2.00 (1.95, 2.05)	2.0	2.10 (2.05, 2.16)	2.00 (1.96, 2.05)	2.01 (1.97, 2.06)	2.0	1.99 (1.94, 2.05)	1.96 (1.90, 2.02)	1.99 (1.95, 2.04)
$M_{A\to B}$	0.2	0.20 (0.19, 0.21)	n/a	0.20 (0.19, 0.21)	n/a	0.02 (0.01, 0.03)	n/a	0.00 (0.00, 0.01)	0.2	0.19 (0.15, 0.23)	n/a	0.20 (0.17, 0.22)
$M_{B\to A}$	n/a	n/a	0.02 (0.00, 0.04)	0.01 (0.00, 0.01)	0.2	n/a	0.20 (0.19, 0.22)	0.20 (0.18, 0.21)	0.2	n/a	0.25 (0.14, 0.35)	0.20 (0.16, 0.24)

Note.— Values of τ and θ are multiplied by 10^3. Results for all data sizes (L = 250, 1000, 4000 loci) are shown in figure 5**h**. 'n/a' indicates that the parameter does not exist in a given model.

Table S4: Bayes factors for comparing models of gene flow for the spruce dataset (fig. 6), calculated using thermodynamic integration and Savage-Dickey density ratio

		Thermodynamic integration		Savage-Dickey density ratio	
Model 1	Model 2	Raw B_{12}	Adjusted B_{12}	$\epsilon = 10^{-4}$	$\epsilon = 10^{-5}$
(i) Pulse gene flow (MSC-I) versus continuous migration (MSC-M)					
MSC-I: B (linked θ)	MSC-M: IM	$e^{34.0}$	$e^{33.9}$	n/a	n/a
MSC-I: B (linked θ)	MSC-M: IIM	$e^{34.2}$	$e^{33.6}$	n/a	n/a
MSC-I: B (linked θ)	MSC-M: SC	$e^{33.6}$	$e^{33.1}$	n/a	n/a
(ii) Constraint on population sizes ($\theta_D = \theta_W$)					
MSC-I: B (linked θ)	MSC-I: B (different θ)	0.04	1.65	n/a	n/a
(iii) Simultaneous contribution from both parents ($\tau_D = \tau_E$, hybrid speciation)					
MSC-I: B (linked θ)	MSC-I: C (linked θ)	6.05	0.07	0.45	0.49
MSC-I: B (different θ)	MSC-I: C (different θ)	4.48	0.25	0.65	3.56

Note.— The Bayes factor B_{12} represents the evidence in favour of model 1 against model 2. We use a cutoff of 1%, with $B_{12} > 100$ or $\log(B_{12}) > \log(100) = 4.61$ representing strong support for model 1 while $B_{12} < 0.01$ representing strong support for model 2. The adjusted value was obtained by smoothing the power posteriors (see Methods). The Savage-Dickey density ratio is applicable to nested models only.

Table S5: Posterior means and 95% HPD intervals (in parentheses) for parameters for the spruce dataset obtained from five models in figure 6.

	MSC-I: B (linked θ)	MSC-I: B (different θ)	MSC-I: C (linked θ)	MSC-I: C (different θ)	MSC-M: IM	MSC-M: IIM	MSC-M: SC
θ_W	8.65 (6.90, 10.44)	6.31 (4.32, 8.59)	8.82 (7.09, 10.63)	7.03 (5.08, 9.05)	7.96 (6.17, 9.86)	7.86 (6.04, 9.65)	7.90 (6.12, 9.73)
θ_P	21.95 (16.93, 27.08)	25.27 (17.51, 33.71)	24.36 (19.13, 29.69)	21.69 (16.45, 26.97)	28.06 (21.99, 34.32)	28.29 (22.26, 34.70)	28.11 (22.23, 34.48)
θ_L	13.31 (10.38, 16.32)	13.01 (10.01, 16.12)	15.42 (12.84, 18.21)	12.26 (9.50, 15.17)	12.38 (9.81, 14.95)	12.37 (9.80, 14.93)	12.46 (9.88, 15.04)
θ_R	6.47 (4.41, 8.67)	6.10 (4.11, 8.16)	6.36 (4.32, 8.58)	6.17 (4.19, 8.29)	10.10 (7.33, 12.79)	10.40 (7.72, 13.21)	10.21 (7.42, 13.02)
θ_D	n/a	15.74 (8.55, 23.76)	n/a	13.74 (7.83, 20.35)	n/a	n/a	n/a
θ_E	25.02 (13.99, 37.42)	24.74 (14.24, 36.51)	n/a	26.85 (16.09, 38.68)	23.76 (5.23, 43.51)	22.65 (3.94, 42.24)	22.80 (4.51, 42.44)
θ_H	n/a	6.78 (0.61, 14.47)	n/a	n/a	n/a	n/a	n/a
τ_R	1.12 (0.91, 1.33)	1.20 (0.98, 1.42)	1.12 (0.91, 1.33)	1.19 (0.98, 1.41)	0.68 (0.54, 0.83)	0.66 (0.53, 0.80)	0.67 (0.53, 0.82)
τ_E	0.48 (0.36, 0.60)	0.43 (0.31, 0.56)	0.45 (0.38, 0.52)	0.37 (0.29, 0.45)	0.54 (0.42, 0.66)	0.54 (0.42, 0.64)	0.54 (0.42, 0.65)
τ_D	0.34 (0.22, 0.45)	0.27 (0.17, 0.37)	0.45 (0.38, 0.52)	0.37 (0.29, 0.45)	n/a	n/a	n/a
τ_p	n/a	n/a	n/a	n/a	n/a	0.13 (0.00, 0.47)	0.31 (0.00, 0.54)
φ	0.368 (0.196, 0.564)	0.357 (0.193, 0.528)	0.511 (0.353, 0.663)	0.541 (0.364, 0.713)	n/a	n/a	n/a
M	n/a	n/a	n/a	n/a	0.027 (0.001, 0.061)	0.023 (0.001, 0.056)	0.028 (0.001, 0.065)
φ_0	n/a	n/a	n/a	n/a	0.002 (0.000, 0.005)	0.001 (0.000, 0.004)	0.001 (0.000, 0.004)

Note.— Values of τ and θ are multiplied by 10^3. 'n/a' indicates that the parameter does not exist in the given model. φ_0 is calculated using eq. 4.
